# A unifying theory of physics and biological information through consciousness

**DOI:** 10.1080/19420889.2021.1907910

**Published:** 2021-06-08

**Authors:** Pollard-Wright Holly

**Affiliations:** NCIS, (Independent Research), Battleboro, VT USA

**Keywords:** Physics, quantum mechanics, mind, biological information, consciousness, observing ego

## Abstract

This article represents a transdisciplinary theory that attempts, in a nonmathematical way, to reconcile some contemporary concepts of physics with a novel theory of the mind. It represents a thought experiment that consolidates complexity by melding certain unifying natural science concepts into a coherent reality. The foundations of quantum mechanics and the cosmological mysteries of dark energy, dark matter, and normal matter non-dogmatically explained may be accessible to individuals other than those immersed in mathematical formulas. Through reasoning and models, terms are defined and illustrations provided, further clarifying concepts. In this theory, consciousness represents dynamic differences that come to an end. It exists through interdependent relationships between dark energy, focal points of dark matter (FPDMs), and normal matter with associated states of mind: pure awareness, pure mental state, and mental images state, respectively. Consciousness enables the emergence of an observing ego, a viewpoint that defines conscious events but which is not consciousness in and of itself. For topics described throughout the article, there is a mental and physical aspect that through relationship produces change that makes a difference. In this way, the reader, an ‘observing ego,’ with a human cognitive viewpoint, may bridge the ‘gap’ connecting the mental and physical domains. Although the theory can be developed mathematically in more detail, the main emphasis is to provide an intriguing explanation of how physics melds with ‘mind,’ thus laying the foundation for future explorations into how this theoretical framework of the mind reciprocates with other areas of science.

## Introduction

Arguably, for science to progress in a way that allows scientists to tackle the complex problems that are the reality these days, researchers need to transcend ordinary understanding. Thus, scientific understanding comes through a melding of different explanations from many disciplines. The author’s central claim is that individuals who transcend the ordinary understand many different things, not just one, implying something has to be added into the equation for scientific discovery to move forward. This theory’s tenet is that the mind as a fundamental entity without beginning or end with infinite potential is not entirely conceivable. Nevertheless, through infinite potential existing, the mind causes change to occur as the potential energies: dark energy, dark matter as focal points of dark matter (FPDMs), and normal matter. These are potential energies because of their relationship to the mind. Arguably, the mind can be explained in part by an abstract idea [1], the law of conservation of energy: the mind cannot be created or destroyed, but potential energies convert or transform from one form into another. Potential energies are change. Though the mind does not change when it manifests its infinite potential, as a consequence, change occurs. When potential energies convert or transform from one form into another, dynamic differences occur through interdependent relationships. Consciousness exists through these interdependent relationships. The definition of the term consciousness has several meanings. In biomedical science, the terms “conscious state” or “waking state” mean the state of waking consciousness with subjectivity (e.g., responsiveness to questions, commands, and mild pain, by the classical scalp EEG of waking, and by the ability to describe oneself and current events) [2]. As an experimental variable, consciousness refers to the dimension of consciousness versus unconscious brain events in a way that allows the study of brain differences attributable to consciousness [2]. This definition involves a measurable dimension of variation and differs but correlates with the first definition (i.e., waking state) [2]. In this theory, consciousness is a concept that is synonymous with the Universe concept; but the terms are somewhat different qualitatively.

The Universe is a particular sphere of activity of all existing matter and space considered a whole (i.e., the macrocosm and microcosm). Thus, it represents activity through interdependent relationships of potential energies with different states of mind and associated intelligence acting as a substrate and where a thought system occurs as consciousness. Consciousness enables the emergence of ‘mind’ observing ego that, through this viewpoint that is not consciousness itself, conscious events are defined [2]. Arguably, the microcosm and macrocosm are terms created by ‘mind’ observing ego trying to express its intuitions as best possible about the reality it perceives. The emergence of ‘mind’ observing ego is a process that can potentially link different embodied states of mind through consciousness. This definition of ‘mind’ observing ego involves a measurable dimension of variation. For example, the author represents an observing ego that, while thinking like a human, perceives mental content that includes logical and spatial competencies of varying degrees. You are an observing ego in the state of waking consciousness that is currently reading this article. Thus, a concept illustrated that although there is a difference between ‘mind’ observing ego, the author, and ‘mind’ observing ego, the reader, by way of consciousness, these different viewpoints are linked. Through ‘mind’ observing ego, the Universe includes a particular activity, interest, or mental experience (e.g., touch, feel, sense, measurement, or detection). Reality is arguably synonymous with the term thought and broadly categorized as different intangible mental content. Although it ‘feels’ otherwise, reality as thought ultimately implies, other than mentally, it cannot be touched or grasped. Neither the Universe nor consciousness can be isolated from reality as thought; instead, it is a part of a universal reality where nothing has intrinsic existence, and nothing is really ‘produced’ [3]. The origin of the Universe and any other aspects of ‘objects’ emerging in thought are through the perceptions of ‘mind’ observing ego, a viewpoint of consciousness. An individual, is a perceptual designation of ‘mind’ observing ego while believing itself to be a singular embodied entity. Through the viewpoint of ‘mind’ observing ego, the physical and mental sides of reality are brought together. Understanding the phenomena of ‘mind’ observing ego explains how the various particles and forces unify through a human cognitive viewpoint. ‘Mind’ observing ego explains how free constants in the standard model of particle physics are chosen.

This transdisciplinary theory represents an extrapolation of Einstein’s famous equation E = mc^2^, which shows that energy and mass are interchangeable. Accordingly, a squared quantity of energy converted into a particle of matter its mass m will be squared [3]. The question becomes, where does the conversion or change that makes a difference take place? The answer to this question, according to this theory, is through ‘mind’ observing ego, with a human cognitive viewpoint that converts energy into a particle of matter. In this way, the mental and physical domains connect through a viewpoint that perceives with ‘solidity.’ The idea of a tangibly ‘solid’ reality has dominated Western philosophical, religious, and scientific thought for over two thousand years; however, discoveries in physics refute this sort of reality [3]. Specifically, quantum mechanics developed at the beginning of the twentieth century undermined the nineteenth-century classical argument of science that objects had an intrinsic existence governed by well-determined cause and effect [3]. The orthodox interpretation of quantum mechanics called the Copenhagen interpretation put forth by Niels Bohr and many others at the beginning of the 20th century argued that physical quantities only ‘come into existence upon measurement’ [4]. This science that considers the observer as an integral part of the physical situation seems to be in harmony with Eastern thought [4]. This melds well with the religions and philosophy (e.g., Buddhism) that emerged in the world of appearances roughly 2,500 years ago. Accordingly, phenomena lack any autonomous or permanent existence, and ‘emptiness’ is the ultimate nature. The term ‘emptiness’ is not a void or absence of phenomena nor a form of nihilism or the belief in nothingness, nor does it correspond to nonexistence [3]. Emptiness refers to understanding the existence, or nonexistence, of so-called indivisible particles of matter and phenomena as essential unreality [3]. With regards to awareness, there is a difference in interpretation. The eastern approach is only quantitative and where the subject/object separation is at least in part an illusion of an individual’s senses as opposed to a qualitative difference in western thought [4].

**Figure 1.**
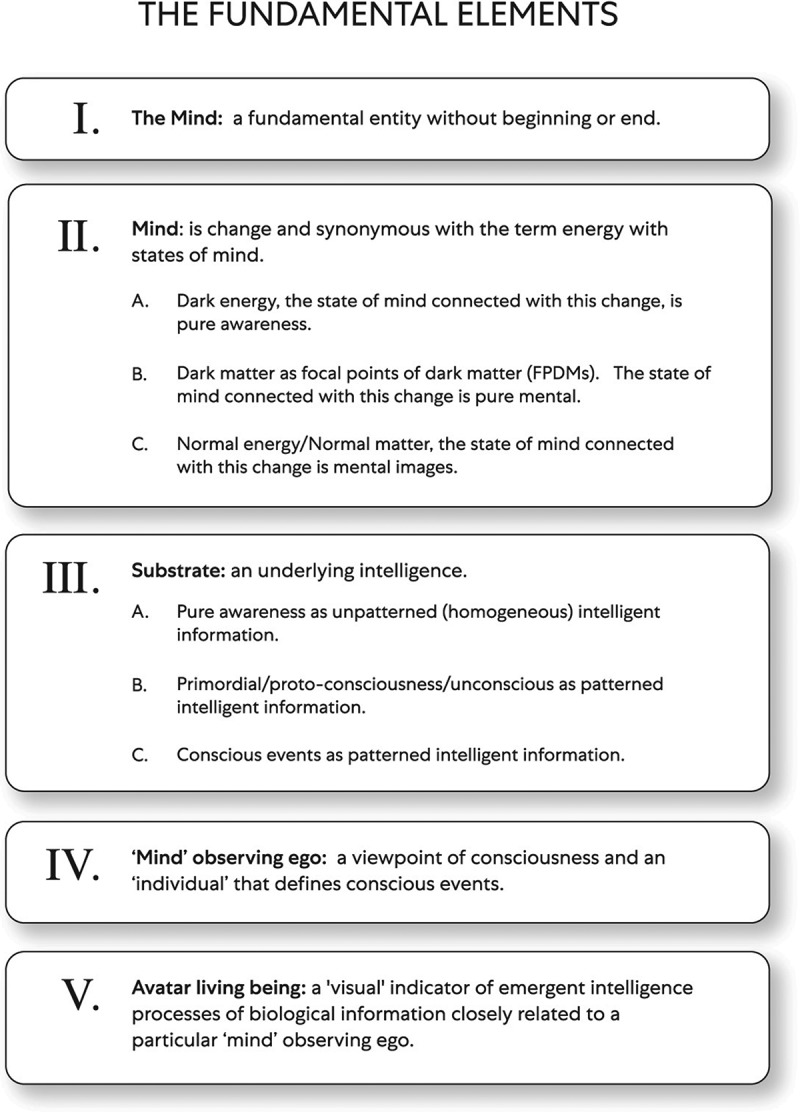
The fundamental elements

This theory was created through a transdisciplinary process where instead of trying to create a new one, the author looked for aspects of theories, including peer-reviewed theories that, through similarities, could be integrated. This theory is a transdisciplinary theory because it relates to more than one knowledge branch. Thus represents the melding of mixed concepts integrated through fundamental elements that organize composition principles and where laws and hypotheses are united within a framework. The advantage of the proposed transdisciplinary theory with these fundamental elements is that action is simplified without complex mechanisms. In this theory, the Universe and consciousness occur through an interplay between these fundamental elements (see Figure 1):

The Universe/consciousness evolves through the relationship between the fundamental elements.

Fundamental Elements

I. The mind: a fundamental entity without beginning or end, generally regarded as more extensive than the ego or observation act. When used, the mind is a term that is synonymous with the terms unending, lasting, or permanent. The mind is the origin for everything that includes change with and without a pattern, states of mind, underlying intelligence as a substrate, innumerable conscious observers, and ‘objects’ of mental content such as living beings ultimately.

..

II. Mind: is change and synonymous with the term energy. Change may be conceptualized in many ways and exists fundamentally through infinite potential and transformation processes. When transformation occurs, change exists that may be without a pattern. Thus there are instances of transformation where change exists, forming no discernible patterns, and change is patternless. This type of change represents a certain continuity without a characteristic or trait by which an individual can describe it by observation, measurement, or combination. Change can also be patterned and described by oscillatory motion. Oscillation is a physical process that goes through many cycles of identical behavior that repeats after a fixed time interval [5]. Some examples, vibrations of plucked strings of a musical instrument [4] or the activity of thousands of neurons firing simultaneously [6]. As related to this theory, oscillation is a process understood through the oscillatory motion of two types of dark energy strings (i.e., open strings and closed strings). The author chose two types of strings derived from string theory with an oscillatory motion to represent change with a pattern because this motion is used throughout science to study many phenomena – for example, electromagnetic waves, alternating currents, and molecules. Moreover, strings of two types as oscillatory objects can bridge the macrocosm and microcosm events gap. However, the two types of strings in this article are described in such a way that an individual may picture a string oscillating in different dimensions. This description differs from a mathematical string’s energy vibrations as a one-dimension construct with length but no thickness, perhaps in the Planck Length order, as measured in 4D space-time.

III. Substrate: an underlying intelligence that consists of conscious events’ intelligence, primordial/proto-consciousness/unconscious intelligence, and pure awareness intelligence. An essential concept within this theory is understanding energies with states of mind that convert from one form into another. The conversion of energies occurs through interaction and relationship, thus transforming states of mind associated with different substrate intelligence.

II/III In this article’s discussion section, change, states of mind, and substrate are concepts merged to create a combined fundamental element category as Mind: Change/Energy/States of Mind/Substrate. In this way, the mental and physical domains of the Universe/consciousness concept are consistently correlated:

1. Dark energy is opaque, and the state of mind connected with this change is pure awareness. The change that makes a difference in this context is through a substrate as homogeneous intelligent information. In this theory, the term ‘dark’ is associated with pure awareness with mysterious qualities unmeasurable qualitatively, but the key to understanding why aspects of the Universe’s origins emerge in consciousness with a ‘mystery to solve.’ Although not entirely conceivable, the mental state of pure awareness has been studied and described in religion and philosophy (e.g. Buddhism). Through techniques described roughly 2,500 years ago, beneficial contributions to scientific discovery involving the mental and physical domains arguably can and have been made. Arguably meditative techniques represent a ‘contemplative science.’

2. Dark matter is opaque and, according to this theory, has oscillating dark energy strings of two types: open strings and closed strings. The state of mind connected with focal points of dark matter (FPDMs), thus change, is pure mental. The change that makes a difference in this context is through a substrate as patterned primordial/proto–consciousness/unconscious intelligent information.

3. Normal matter is visible and, according to this theory, exists because invisible normal energy transforms into normal matter. The state of mind connected with normal matter, thus change, is mental images. The change that makes a difference in this context is through a substrate as intelligent information of patterned conscious events.

IV. ‘Mind’ observing ego: a viewpoint of consciousness that defines conscious events but is not consciousness in and of itself [2]. The observing ego is a concept used throughout scientific literature such as physics [7], neurology [2], and psychology (William James 1842-1910). The viewpoint of ‘mind’ observing ego is synonymous in this theory with the more familiar term individual that through interdependent embodied relationships brings the physical and mental sides of reality together when defining conscious events.

V. Avatar living: a ‘visual’ indicator of emergent intelligence processes of biological information. When ‘mind’ observing ego experiences conceptual mental content (e.g., conceptual thoughts or memory) later in cognitive construction, it often includes a Avatar living beings with distinctive characteristics such as a brain (e.g., invertebrates, mammals, birds, amphibians, reptiles, and fishes) and those without a brain (e.g., slime mold and plants). A living organism is a requirement for the study of physiology and behavior through biology, and thus Avatar living being is a term used to bridge the gap with information technology.

‘Relative truth’ is defined here as the usual way ‘mind’ observing ego, with a human cognitive viewpoint, perceives the world of appearances. It is ‘mind’ observing ego with the perception of an individual that observes phenomena. It is ‘mind’ observing ego that is the perceiver of all kinds of mental content that includes (with a human cognitive viewpoint) the perception that the Universe is expanding derived from relative observations of temperature fluctuations of the cosmic background radiation indicating the existence of mysterious ‘dark energy.’ With conceptual mental content, ‘mind’ observing ego may understand reality: the state of things, including physics laws and many basic scientific concepts, albeit with ambiguity. For example, the Universe and its expansion are related to dark energy. Approximately 68% of the Universe is dark energy. Dark matter makes up about 27% [8]. The rest that includes everything in the world of appearances is normal matter. Accordingly, normal matter adds up to less than 5% of the Universe [8]. In classical physics and general chemistry, matter is any substance with mass and takes up space by having volume. Dark matter, dark energy, and distribution in space are inferred from observations of normal matter using physics’s known laws with discrepancies of motion. Under the influence of its gravity, the motion calculated for normal matter does not agree with its observed motion [5]. This discrepancy can be explained through the Universe/consciousness concept and fundamental elements.

As previously mentioned, pure awareness, the state of mind associated with dark energy, represents an underlying pure awareness intelligence substrate. This intelligence is devoid of motion differences. Thus, pure awareness, a state of mind dark energy, represents a change fundamentally closest to representing the mind without beginning or end. ‘Mind’ observing ego, with a human cognitive viewpoint, can define reality and infer through physics laws measurement, matter, energy, and motion. However, what it cannot do with thought is describe pure awareness, the state of mind dark energy devoid of motion differences, through a qualitative difference (i.e., observation, measurement, or a combination). Although the perception of ‘mind’ observing ego through cognition can bridge the gap between the mental and physical domains, this intelligence is different from and not a substitute for pure awareness intelligence. Instead, both coexist and represent a vital relationship with regards to the Universe/consciousness concept. This relationship explains why the perception of ‘mind’ observing ego, with a human cognitive viewpoint ‘thinking physics,’ can define reality, albeit where a lack of compatibility between two or more ‘facts’ exists, for example, discrepancies of calculated versus observed motion.

This theory’s framework consists of fundamental elements and terminology used in this article’s discussion section is meant to bridge science and philosophy. Included in the discussion section are descriptions and abstractions not meant to disclose the real essence of phenomena. Attributes from theories have multiple meanings and include energy, matter, space, and structure, resulting from melding concepts on a small number of phenomena to construct a coherent picture of the Universe. Through the ‘Algorithm of Fundamental Elements,’ an origin story of the Universe is depicted that includes concepts derived from scientific theories. The italicized text represents statements that are matched with a discussion. In this article’s discussion section, phenomena are intended for ‘mind’ observing ego, i.e., the reader, to perceive images that may help put together diverse conceptual mental content into a coherent and logical scheme.

## Discussion

*The Origin of the Universe is a science story about what happens when the mind’s infinite potential manifests change.*

The article’s discussion section includes a model and nondogmatic account and ‘Origin Story of the Universe.’ Origin applies to potential energies. Thus a change that occurred as a consequence when the infinite potential of the mind manifested from which ultimately consciousness is derived, and the causes operating before the change came into being. As dynamic differences in this theory, consciousness occurs through the interplay of innumerable embodied energies (i.e., embodied states of mind): mind normal matter and mind FPDMs with respective states of mind mental images and pure mental. The substrate for embodied states of mind is pure awareness, the state of mind associated with dark energy. Correspondingly the three states of mind have emergent properties representing intelligence: conscious events’ intelligence, primordial/proto-consciousness/unconscious intelligence, and pure awareness intelligence, respectively. These interdependent relationships’ causes have no beginning, are unlimited, and cannot be reduced to a single prime cause. Instead, the mind without a principle of organization, as a fundamental entity without beginning or end through infinite potential existing, caused change. Change as potential energies that convert or transform through embodied interdependent relationships modifies how consciousness evolves.

According to this theory, in general, dark energy, FPDMs, and normal matter with copresent states pure awareness, pure mental, and mental images, respectively, are change. Mind dark energy in this theory ‘morphs’ through two types of dark energy strings. Thus, a dynamic change occurs by transforming a potential energy’s appearance and character, and from this emerges mind FPDMs. As part of this process, pure awareness transforms into a pure mental state: a quality of ‘knowing.’ Pure mental, the state associated with dark matter as FPDMs, represents an underlying primordial/proto consciousness/unconscious intelligence. This intelligence has motion differences through the oscillatory motion of strings. Mind FPDMs with the state of pure mental through embodied relationships is the dynamic change that makes ‘knowing’ measurements of stimuli to map them constructively. In turn, this gives rise to ‘mind’ observing ego. Cognition, the mental state associated with mind ‘observing ego,’ represents an underlying cognitive intelligence. This intelligence has motion differences through the motion of mental content. These events are interdependent, implying a dynamic change that makes innumerable differences occur through the crucial aspect of transformative relationships.

I The mind: The fundamental entity

*The mind without a beginning or end with infinite potential is not entirely conceivable.*

**Figure 2.**
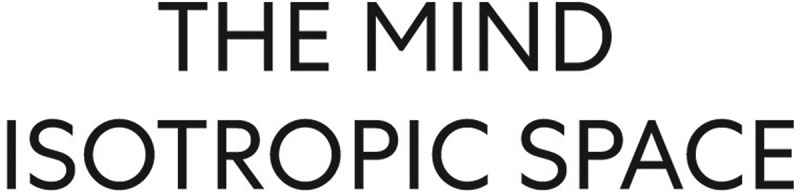
The mind and inconceivable reality

The mind without a beginning or end with infinite potential is not entirely conceivable but envisioned in this theory as isotropic space, which is not entirely ‘thinkable’ and thus causes an incomplete ‘Origin Story of the Universe.’ Isotropic space is a nondogmatic depiction of the mind with immeasurable potential. However, this quality is not entirely ‘graspable’ through conceptual mental content because it lacks a discernable pattern (see Figure 2).

Caption: Isotropic Space

II/III. Mind: Change/Energy/States of Mind/ Substrate

*Because the mind exists without beginning or end, unobservable reality existed before the ‘Big Bang.’*

**Figure 3.**
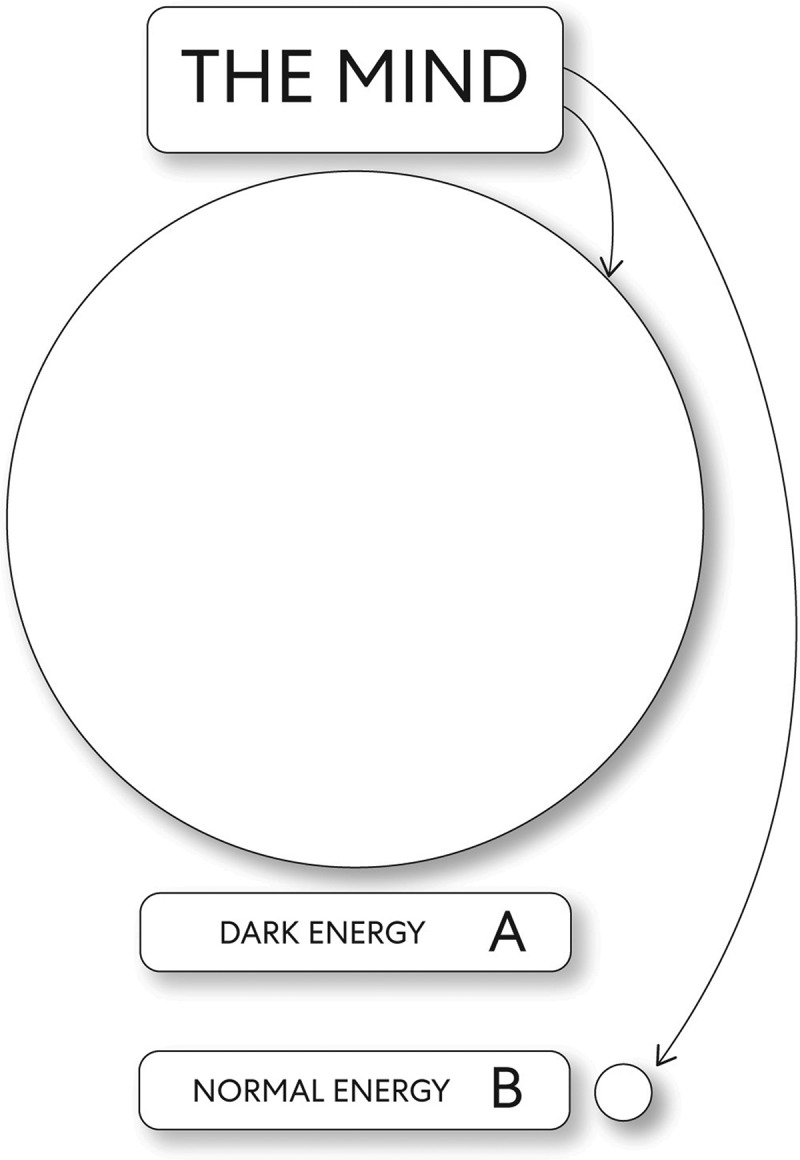
The mind, and mind potential energies: unobservable reality manifests

What follows inconceivable reality is the mind without beginning or end through its infinite potential caused change to occur. In this case, the change that makes a difference occurred through the potential energies: dark energy and normal energy. However, mind was in a much smaller form as normal energy than dark energy. Thus, mind dark energy was a much larger mass density than mind normal energy, and because of this, there was a potential that normal energy would be ‘swallowed up’ if there was a relationship between these two forms of energy. A dynamic change as energies with potential prevented from creating a meaningful relationship implies an essential concept about the functioning of the Universe (see Figure 3). An essential aspect of the Universe’s origins is interdependence, which is not just a simple interaction between phenomena [3]. Instead, a relationship is a precondition, and as related to this scenario, a relationship was needed before the copresent state of mind, mental images get distinctly expressed.

A. Dark energy B. Normal energy

In this part of the story, mind normal energy existed, but in an indistinct state of mind: mental images as the state of mind normal energy although not expressed. However, mind dark energy exists, and a copresent state of pure awareness was distinctly expressed: as change devoid of motion differences. Thus a dynamic change fundamentally closest to representing the mind without beginning or end but without a characteristic or trait by which an individual can describe it by observation, measurement, or a combination. The present state of pure awareness is distinctly expressed because mind dark energy is recognizably different from something else; mind normal energy of a similar type. A similar type here refers to a type of change when the mind’s infinite potential manifested; this caused dark energy and normal energy to emerge as two non-interacting energies. Energies with potential are the dynamic change that occurred through which ‘unobservable reality’ and a macrocosm manifested.

**Figure 4.**
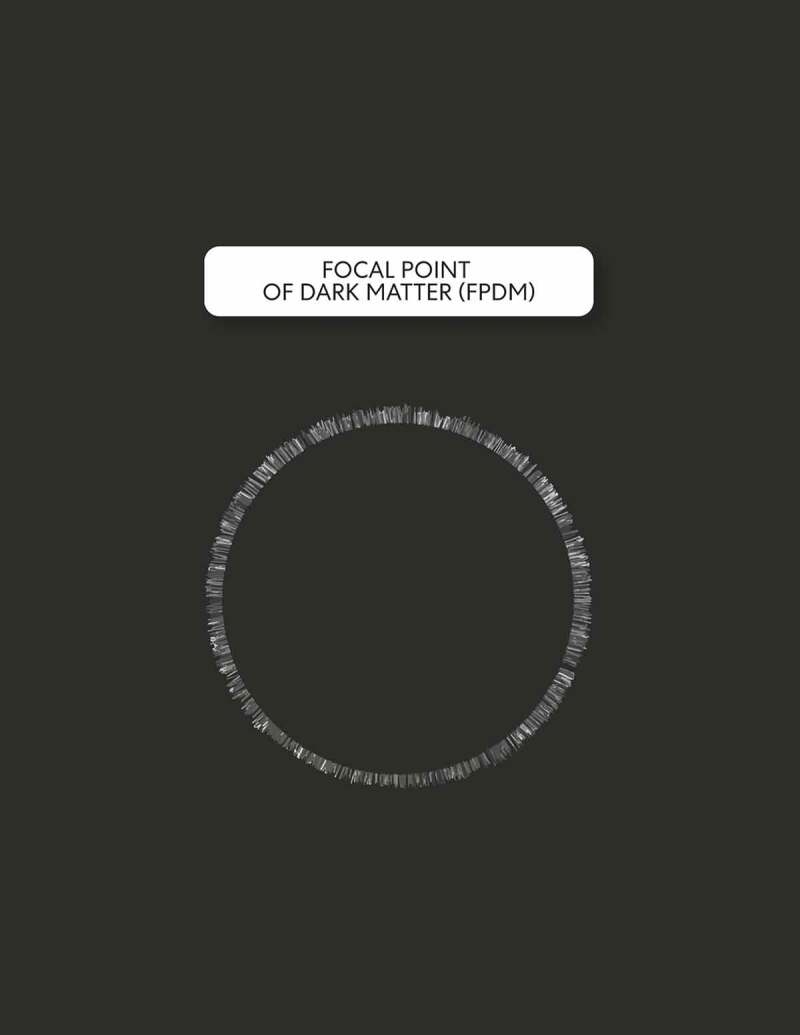
Mind: A focal point of dark matter (FPDM)

Next in the story mind dark energy morphed through dark energy strings of two types: open strings and closed strings. Both open and closed string types are filaments of dark energy, which oscillate and create a definite pattern, as change that makes a difference. Thus pure awareness as the state of mind dark energy transformed. Through the arrangement of open and closed oscillating strings, perfect geometric shapes like circles manifested. Thus, in the macrocosm, emerged mind FPDMs: circles of compacted multi-dimensional space demarcated through the oscillatory motion of open and closed dark energy strings [9] (see Figure 4).

Mind a FPDM emerges in the macrocosm when mind dark energy morphs through the oscillatory motion of two types of strings. In this figure, open strings and closed strings appear the same, and with a bit of imagination on the part of ‘mind’ observing ego, the reader, envisioned with movement.

In this theory, pure mental states of mind FPDMs represent an underlying intelligence with motion differences through strings’ oscillatory motion. This relationship is depicted through an ‘object,’ the conceptual image of a round two-dimensional shape (i.e., a circle) [10].

**Figure 5.**
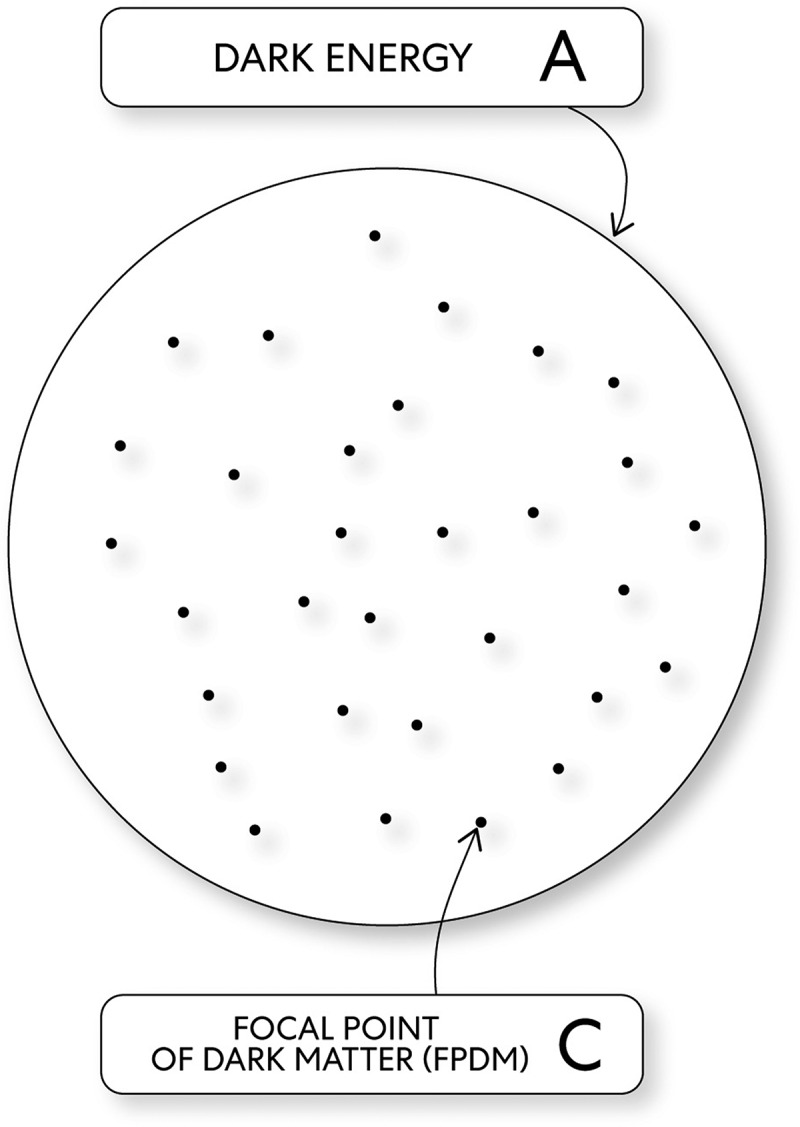
Mind dark energy & mind FPDMs

Mind as each focal point of dark matter (FPDM) has a position on the Universe’s dark energy substrate related to all other FPDMs. The open and closed strings of mind FPDMs share the same ground state when oscillating with minimal energy and the lowest vibration mode [11] (see Figure 5). Dynamic change with limits, consequence, and duality related to pure awareness’s transformative properties exist through the oscillatory motion of two types of strings; open strings and closed strings. The emergence of mind FPDMs brings about a change in universal local properties. A consequence that causes the transfer of an amount of concentrated dark energy [12]. Thus, mind FPDMs with pure mental state increased through concentrated pure awareness, and this caused some closed strings to change their ground state’s oscillations to their first excited state. In this way, mind a FPDM generates momentum with the first excited state of some closed strings and, through gravity, an invisible consequence pulls mind normal energy toward it.

A. Dark Energy, C. FPDMs

**Figure 6.**
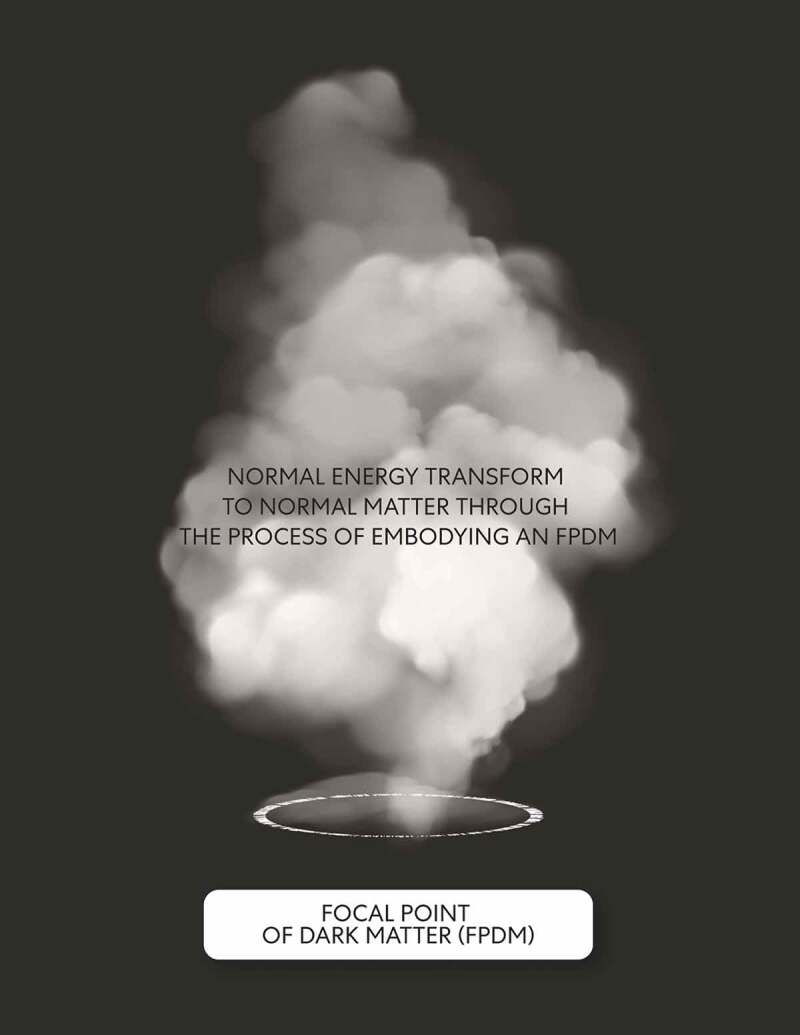
Process of embodiment

**Figure 7.**
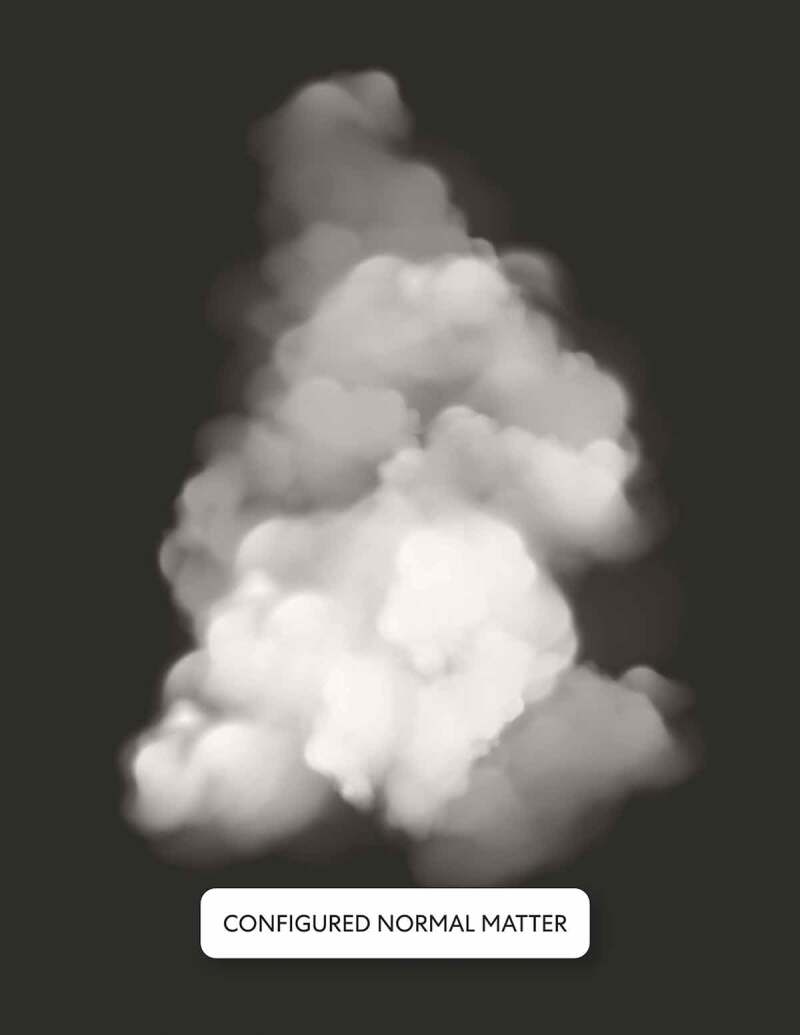
Embodied

**Figure 8.**
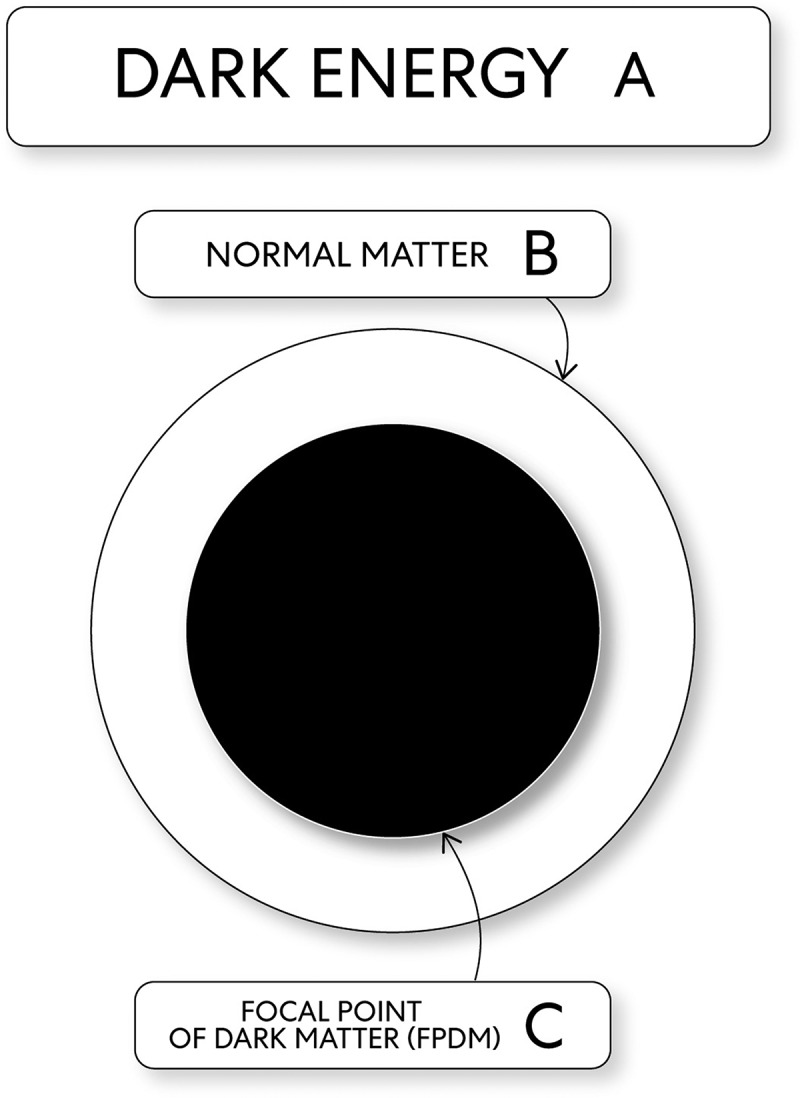
Cross-section view of embodiment

Thus, a transformative mechanism is described through closed strings of mind FPDMs and where the non-local gravitational field of mind dark energy in the macrocosm becomes local [13]. The transformed mind normal energy into mind normal matter is this event’s outcome and a process that occurs while embodying mind FPDMs. The act of embodying mind FPDMs caused the transformation of mind: mental images as the state of mind normal energy although not expressed into mental images as the state of mind normal matter where mental images get expressed (see Figure 6, Figure 7 and Figure 8). This transformation implies that the fundamental difference between normal energy and normal matter is through the mental images state. The state of mind associated with normal matter represents an underlying conscious events’ intelligence. According to this model, because of the number of FPDMs, the existence of mind normal energy, is brief and undetectable. Thus most commonly in the form of normal matter that embodies mind FPDMs.

The closed strings of mind a FPDM oscillate in the excited state; the outcome is a transformation of mind normal energy into mind normal matter.

Mind normal matter is configured and embodies mind a FPDM (not seen in this figure).

In this figure mind a FPDM is exposed by making a straight mental cut through the embodiment frame work of the configured mind normal matter.

Gravity in the macrocosm is not a force but rather a consequence of an interdependent relationship. This illustrates that things in the Universe do exist somehow, but nothing exists on its own [3]. In this model, when mind FPDMs becomes embodied by mind normal matter, the conceptual designation related to the story’s location changes. Rather than in the macrocosm, embodied states of mind are located in the microcosm.

*The microcosm represents a set of circumstances when mind FPDMs becomes embodied by mind normal matter, and when the conceptual designation related to the story’s location changes, a symbolic ‘Big Bang’ occurs.*

In the microcosm, each mind a FPDM embodied is folded into a discrete volume of mind normal matter. The microcosm represents a set of circumstances that ‘encapsulates’ the features of something larger (i.e., dynamical macrocosm/relationships between energies with potential and a dynamic change that makes a difference) [14]. The Big Bang in this model is merely an abstraction that occurs through a process when theories are melded to construct a coherent picture of interdependent embodied relationships.

*The circumstances occurring in the microcosm can be envisioned through the concepts of a ‘body,’ ‘cosmic brain,’ and framework.*

**Figure 9.**
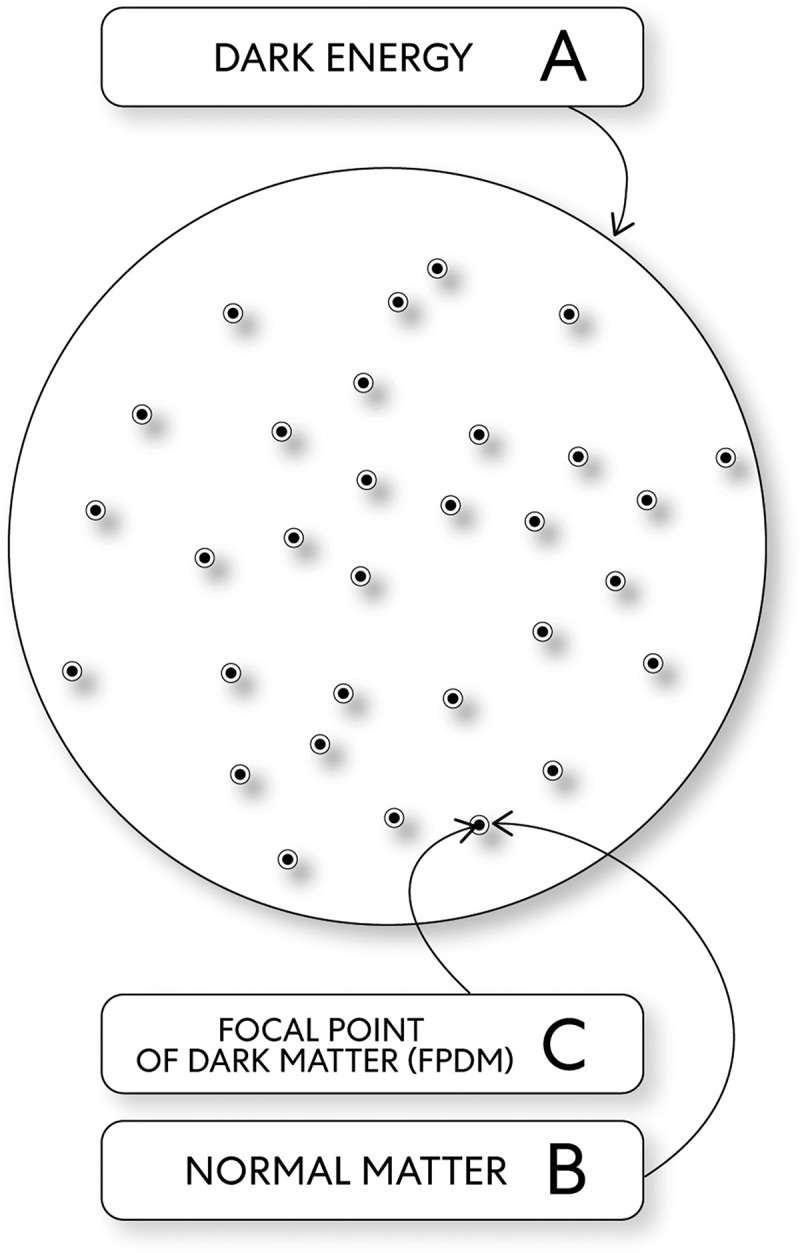
The structure of dramatic change: mind (potential energies)

1. ‘Body’: pure awareness as the state of mind dark energy acts as a substrate of homogeneous intelligent information underlying embodied states of mind. This substrate represents a network (i.e., interoceptive network) that connects innumerable embodied energies that condition each other. Mind dark energy with pure awareness intelligence ‘orchestrates’ the merging of a pair or a group of embodied relationships regardless of distance and perhaps in a way that is ‘spooky’ because communication between them is naturally simultaneous [15].

2. ‘Cosmic Brain’: pure mental as the state of mind FPDMs through dark energy strings of two types; open strings and closed strings makes ‘knowing’ measurements of stimuli to map them constructively. According to this model, rather than thinking of a brain as an organ of soft nervous tissue in vertebrates’ skulls, mind a FPDM in an embodied relationship is the coordinating center of sensation and intellectual activity. Thus, phenomena emerge as ‘objects’ of conceptual mental content preceded by primordial/proto-consciousness/unconscious intelligence events.

3. Framework: mental images as the state of mind normal matter through an event’s outcome, where mental images get expressed acts as the essential supporting structure for embodied states of mind (see Figure 9).

A. Dark Energy. B. Normal Matter. C. A FPDM

*Consciousness occurs in the microcosm and is due to a cooperative condition*

According to this model, consciousness occurs in the microcosm due to a cooperative condition. The origin story of the Universe continues, where gravity is too weak in the microcosm to configure mind normal matter that embodies mind FPDMs. In the microcosm, the fundamental force associated with configuring embodied relationships is electric and magnetic fields (i.e. electromagnetic) generated through the open and closed strings’ oscillatory motion of mind FPDMs. Electromagnetism, as a cooperative condition, occurs where mind FPDMs embodied in the microcosm while interacting with mind normal matter have open and closed strings; thus, a transformative mechanism through oscillatory motion as contact forces. These forces shape mental images as the state of mind normal matter bends and gives way through stresses placed upon integrated frameworks that turn ‘outside-in’ or ‘inside-out’ [16].

Over billions of years in this model and’ origin story,’ electromagnetism played a part in interdependent relationships by causing mind normal matter to fold, and through contact forces, increased available surface area allowing for more complex circuitry. As previously mentioned, this section of the article intends to provide descriptions. With this idea, the term circuitry depicts mind normal matter in an arrangement that acts as a more or less complicated framework for embodied states of mind. Mind normal matter began configuring more complexly conceptualized in this way: mind normal matter convoluted with columns where ‘minute circuits’ function as processors perhaps with billions of components. Innumerable configurations of embodied states of mind exist in the microcosm, and those frameworks with intricate functions that get duplicated repeatedly are more complex.

What is similar between frameworks of configured mind normal matter and mind FPDMs as ‘cosmic brains’ occurs through electromagnetic radiation (EMR): waves of energy traveling the speed of light through electric and magnetic fields that vary simultaneously. Dynamic change as the mental images state associated with mind normal matter represents an underlying conscious events’ intelligence. This intelligence has differences through the oscillatory motion of circuitry. Conscious events’ intelligence generated by the active circuitry of mind normal matter transmits through EMR to mind FPDMs. When mind FPDMs receive information from mind normal matter, it is transformed through the oscillatory motion of strings in the excited state.

Similarly, dynamic change as a pure mental state associated with mind FPDMs represents an underlying primordial/proto consciousness/unconscious intelligence. This intelligence has differences through the oscillatory motion of open and closed strings. Primordial/proto-consciousness/unconscious intelligence transmits through EMR to mind normal matter. When mind normal matter receives information from mind FPDMs, it is transformed through active circuitry. Thus, mind FPDMs and mind normal matter are in a communicative relationship. Correspondingly they influence each other during every instant, with a delay occurring when information is sent and received.

*For a cooperative condition to occur, the strings of mind FPDMs need to change from the ground state to the excited state.*

According to this model, the exact number of open and closed strings of mind FPDMs attached to a dark energy circle (a membrane of compacted multi-dimensional space) is unknown. However, strings’ arrangement is as follows: there are strings open and closed that form an inner layer and strings open and closed that form an outer layer. Strings remain oscillating in their ground state; open and closed strings share the same ground-state until enlisted. The term enlisted is defined here as an occurrence where a string’s oscillatory motion changes from a ground state/minimal energy and the lowest vibration mode to an excited state/higher energy than the ground state. Mind FPDMs enlist the number of open and closed strings needed on a momentary basis.

With a copresent state of pure mental and emergent properties representing primordial/proto-consciousness/unconscious intelligence, mind FPDMs are aware of the oscillatory motion of strings and the number needed to transform any momentary flow of information received from mind normal matter. Accordingly, received through EMR, not every string of mind FPDMs is enlisted to transform intelligent information of patterned conscious events generated by the active circuitry of mind normal matter. Instead, on a momentary basis, some strings remain in their ground state, and in this state, one end of the string is attached to compacted multi-dimensional space (i.e. dark energy circle), and one end is open. Enlisted strings open and closed can oscillate in different patterns, and thus mind FPDMs are reactive to signals through EMR received from mind normal matter. In this context, information is an emergent intelligence process that includes oscillatory motion of dark energy strings acting as dynamic change through signals [17].

*Information received from mind normal matter gets transformed similarly through the oscillatory motion of enlisted open and closed strings that form an inner layer.*

When enlisted to transform information, open and closed strings that form an inner layer reactively change oscillatory motion from the ground state to the excited state. The circle of compacted multi-dimensional space functions similarly to a frictionless one-dimensional ‘object.’ Once enlisted, both ends of a string located on the inner layer slide up and down as if doing so within a ‘frictionless hoop.’ However, the curvature (i.e., gradient or differential) of the string is constrained at the endpoints. The oscillatory motion of enlisted open and closed strings that form an inner layer fundamentally transform signals received through EMR. Thus ‘fundamentally’ implies that information received from mind normal matter in primary respects gets transformed similarly. This activity then connects to strings open and closed on the outer layer. Dynamic change as the oscillatory motion with potential between the strings forming an inner layer gets propagated in many different ways by the outer layer of strings oscillatory motion.

*Enlisted open and closed strings that form an outer layer reactively change oscillatory motion from the ground state to the excited state and thus transform information received and when they are triggered.’*

Enlisted open and closed strings that form an outer layer reactively change oscillatory motion from the ground state to the excited state and transform information when:

A. received as signals from mind normal matter through EMR.

B. triggered by the inner layer of enlisted strings.

Enlisted open strings that form an outer layer change oscillatory motion from the ground state to the excited state by bending and connecting. Thus, the end of the string once free now connects to the circle of compacted multi-dimensional space. Enlisted open strings now have fixed endpoints with both ends attached to a dark energy circle of mind a FPDM. The endpoints’ conditions determine the frequencies at which these open strings may oscillate in the excited state. However, open strings with fixed endpoints are temporary, and the end of the string once free fixed to the circle of compacted multi-dimensional space eventually disconnects. When this happens, the string recoils and configures as the previous ground state, one end attached to the circle of compacted multi-dimensional space and the other end free.

Enlisted closed strings forming an outer layer change oscillatory motion from the ground state to the excited state by bending so that their free end ‘taps in’ to the circle of compacted multi-dimensional space. The end of a closed string then penetrates and whirls into a dark energy circle. Although the end of a closed string may not be ‘seen’ whirling beneath the surface of compacted multi-dimensional space, it whirls until it meets and connects with the other end of the string. The ‘tapping in’ and whirling by the end of the closed string functions as a kind of momentum. However, the ‘tapping in’ and connecting by the end of the closed string oscillating in the excited state is temporary, and the end eventually disconnects from the other end of the string. When this happens, the string recoils and configures as the previous ground state, one end attached to the circle of compacted multi-dimensional space and the other end free.

*Through the inner oscillatory string layer, physiological systems regulate, and thus ‘sameness’ is created as a reference point.*

Some signals sent through EMR by mind normal matter get transmitted continuously and similarly. These signals relate to critical physiological processing for a particular embodiment that deviate little from ‘day-to-day.’ Generated by the active circuitry of mind normal matter, change as biological information that signals’ life-sustaining’ patterns transmits through EMR to mind a FPDM. As previously discussed, there is a beginning for a particular embodied state through the oscillatory motion of some closed strings of mind FPDMs. Moreover, through the inner oscillatory string layer, a reference point is created as signals through which physiological systems regulate. When signals that deviate little from ‘day-to-day’ transmit continuously through EMR, the oscillatory motion of enabled strings on the inner layer creates ‘sameness.’ This type of oscillatory motion through signals triggers a wave of activation and ‘sameness’ through a collection of enabled strings’ oscillatory motion on the outer layer.

Triggered by the initial change, it is reasonable to say that some information as it flows through the inner layer of enabled strings to the outer layer represents a more or less complicated system. The propagation of intermediate stages may depend on new intermediate change differences, producing new effects elsewhere [18].

*Gravity in the microcosm is a ‘push force,’ and EM waves pushed toward the center of a dark energy circle create a pattern envisioned as a centralized ‘tiny black hole.’*

A fundamental difference between the EMR generated by mind normal matter and mind a FPDM lies in frequency: how often waves of energy peak and trough in a given second. A continuous two-way interplay between mind a FPDM and mind normal matter through embodied states of mind exists. Embodied relationship dynamics cause information to be transmitted through electromagnetic waves of energy (i.e. EM waves). Through mind a FPDM, EM waves radiate on compacted multi-dimensional space’s dark energy surface and get pushed by gravity. Gravity in the microcosm is a ‘push force’ [19]. EM waves get pushed through gravity toward the center of the dark energy circle. Thus creating a pattern envisioned in this model as a centralized ‘tiny black hole’: A dynamic change pattern present on compacted multi-dimensional space with a centralized shape, as a sphere and spheroidal region.

As relates to physics, a black hole, in general, is a place in space where gravity is strong because matter gets squeezed into a tiny space. This event is thought to take place when a star is dying. In this model, a black hole is a place in an embodied state and relates to the pure mental state of mind a FPDM through a patterned change on a dark energy surface of compacted multi-dimensional space. Every moment marks a beginning through the emergent two-way interplay between mental images, the state of mind normal matter, and mind a FPDM with the pure mental state that pushes this interdependent relationship.

*When pushed toward the ‘tiny black hole,’ EM waves bend and configure into conical EMR that in this model represents the form of consciousness produced through embodied states.*

Consciousness represents dynamic differences through constant change and a particular end. In this model, conical EMR represents consciousness produced through embodied states. Through mind a FPDM , EM waves get pushed by gravity on the surface of the circle of compacted multi-dimensional space. Moreover, the oscillatory motion of enlisted open and closed strings forming an outer layer while attached to the circle of compacted multi-dimensional space generate more EM waves at different frequencies than enlisted open and closed strings that form an inner layer. When pushed toward the ‘tiny black hole,’ EM waves bend and configure into conical EMR. The base of the conical EMR contains the least amount of EM waves. As the EMR gets higher, it widens due to more EM waves being present. Conical-shaped EMR radiates and acts as a space-filling field present within the configured mind normal matter. Thus, incorporating EM field-mediated communication into this model can reframe discussions surrounding consciousness [20].

**Figure 10.**
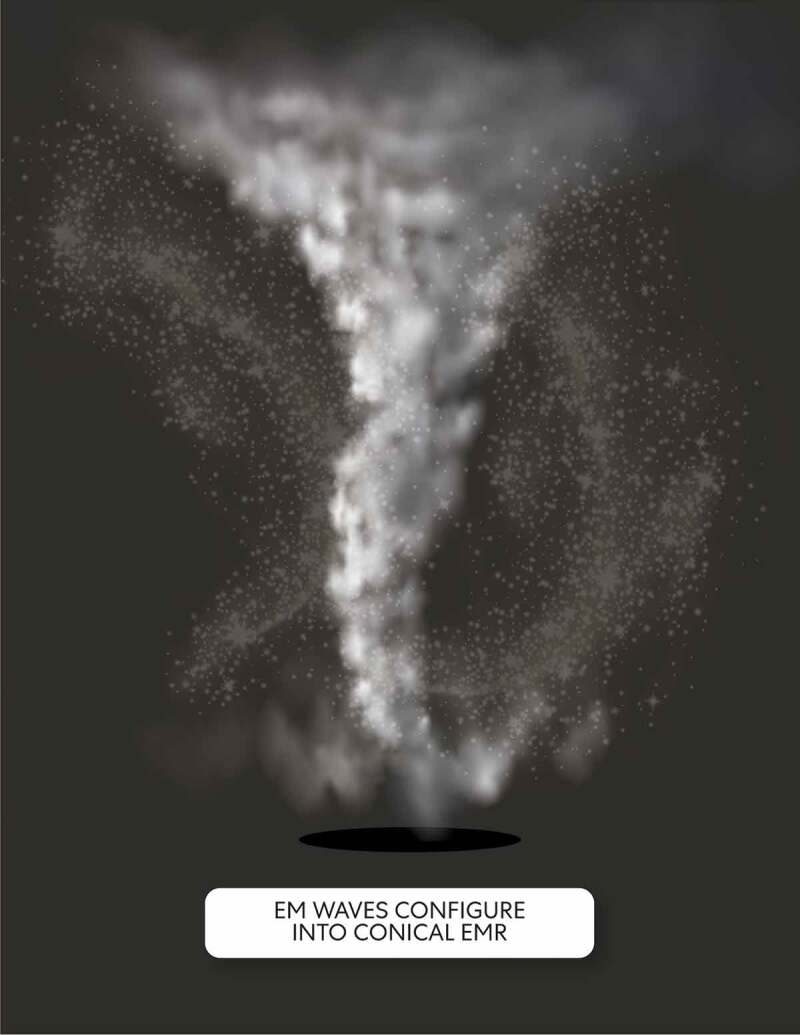
Conical EMR momentarily escapes

The way conical EMR radiates creates a region between the surface of the circle of compacted multi-dimensional space and the base of the EMR. The EM waves of the conical EMR are patterned change as dynamic differences with potential in orbital motion pulled by the sphere of revolution, the centralized spheroidal region. However, because of too much angular momentum, the conical EMR momentarily escapes from being pulled into the tiny black hole.’ This situation implies that consciousness depicted through EMR avoids a particular momentary end through mind a FPDM with the pure mental state (see Figure 10).

Conical EMR (the form of consciousness) and spheroidal region (‘tiny black hole’).

*An underlying intelligence associated with the pure mental state of mind a FPDM transmits from an envisioned ‘tiny black hole’ and in a way that ‘illuminates’ conical EMR.*

**Figure 11.**
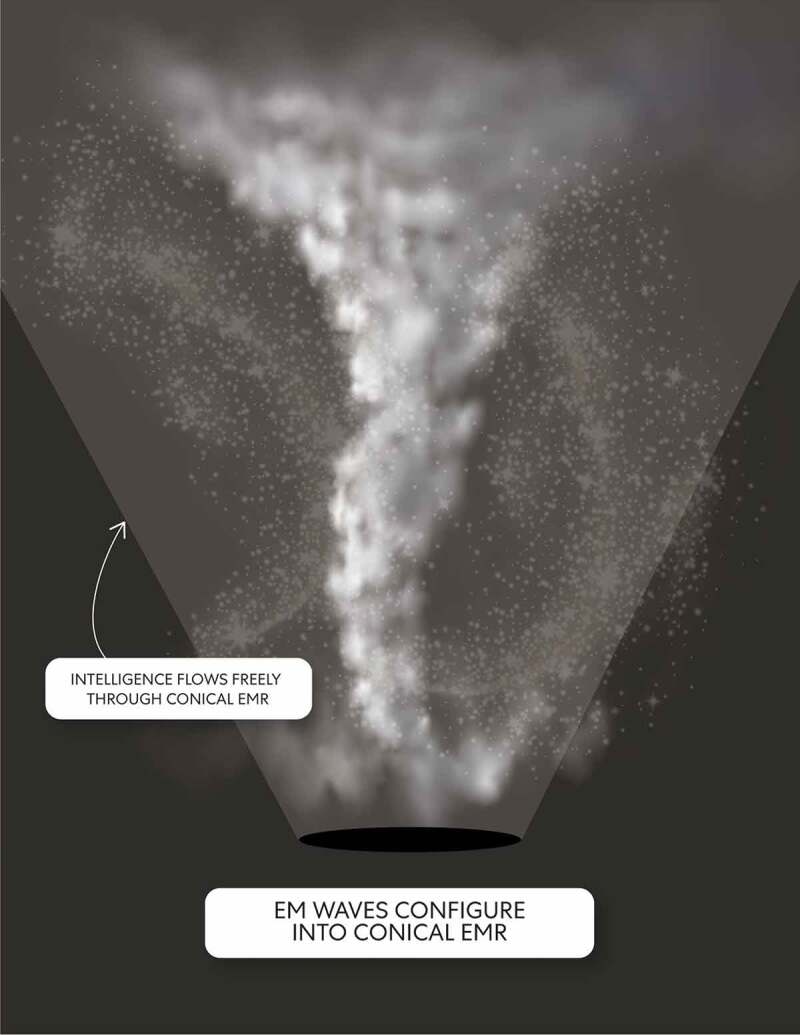
Beam of Intelligence

Each mind FPDMs is the coordinating center of sensation and intellectual activity for an embodied state, however, with limitations implied through the ‘tiny black hole.’ An underlying intelligence associated with the pure mental state of mind a FPDM transmits from a tiny black hole in a specified direction. Accordingly, ‘beaming’ underlying intelligence ‘illuminates’ conical EMR through the dark energy circle surface (see Figure 11). Embodied black holes fundamentally occur through compacted multi-dimensional space when the pure mental state’s force is strong because primordial/proto-consciousness/unconscious intelligence gets squeezed into a tiny space. Accordingly, through the pattern present on the dark energy circle surface, the emergent intelligence of mind a FPDM is constrained through emission from the centralized spheroidal region (i.e., a ‘tiny black hole’).

A pattern is present on the surface of a circle of compacted multi-dimensional space requiring a bit of imagination on the part of ‘mind’ observing ego the reader to be envisioned. An underlying intelligence associated with the pure mental state of mind a FPDM transmits from a tiny black hole in a specified direction. Accordingly, ‘beaming’ underlying primordial/proto-consciousness/unconscious intelligence ‘illuminates’ conical EMR through the dark energy circle surface and a tiny black hole.

Additionally, the oscillatory motion of enabled open and closed strings on the inner and outer layer stimulates mind a FPDM and acts as a control. This control determines the rate at which primordial/proto-consciousness/unconscious intelligence emits through the tiny black hole. The stimulated mind a FPDM uses the force of pure mental state to control the beam’s activity through the tiny black hole center in greater or lesser amount. Mind a FPDM stimulated to varying degrees causes an emergent intelligence emission in the beam’s central region [21]. The correlated amount of intelligence ‘illuminates’ the conical EMR through greater or lesser degrees of stimulated emission.

Mind a FPDM with the pure mental state through this activity represents dynamic change and embodied energy with potential that makes ‘knowing’ measurements of stimuli to map them constructively. Through the oscillatory motion of enabled strings of mind a FPDM, intelligent information as patterned conscious events of mind normal matter gets transformed. EM waves through conical EMR are patterns of dynamic difference that, when detected, cause mind a FPDM to capture small amounts of data to create ‘atoms’; or small amounts of information. Perhaps billions of patterns within EM waves are detected through mind a FPDM that focuses on patterns that differ. In this way, created, ‘atoms’ through encoding are converted when stimuli get mapped [21]. As a statistical entity of sorts mind a FPDM generates explanations for encountered stimuli. However, the ‘explanations,” hypotheses,’ and ‘beliefs’ of mind a FPDM are not consciously held mental states but encoded probability distributions over sensory signals’ hidden causes [22].

*Mental images represent underlying conscious events’ intelligence of mind normal matter that has differences through circuitry’s oscillatory motion.*

As the state of mind associated with normal matter, mental images represent underlying conscious events’ intelligence. This intelligence has differences through the oscillatory motion of circuitry. Thus, a potential difference occurs through EMR and, in this scenario, represents information of patterned conscious events. The dynamic difference through EMR then acts as a processed signal sent to mind a FPDM with a purely mental state. In the first place, through circuitry mind normal matter generates an unexpected stimulus that acts as signals through EMR.

Mind a FPDM functions as a detector of synchrony and an integrator [23] of conscious events’ intelligence. Through EMR, information transmits as waves of energy traveling the speed of light with simultaneously varying electric and magnetic fields. Through strings’ oscillatory motion in the ground state, mind a FPDM detects this information. It reacts to the degree of difference through synchronized oscillations when a string’s oscillatory motion changes from the ground state to the excited state after enlisted. Mind a FPDM is not concerned with the informational content of signals received through EMR, only with the degree of dynamic difference as synchrony through the oscillatory motion of enabled strings. It responds more robustly to synchronized differences as bursts of change rather than random spikes [23].

Mind a FPDM tends to reproduce the synchronization of patterns received through EMR by initiating synchronized oscillatory motion of enabled open and closed strings. Mind a FPDM processes inputs using multiple competitive synchronized oscillatory motions of enabled strings. Thus, it reproduces in a small volume the activity promoting multiple, ﬂuctuating, and differential synchronous oscillations [23]. Reverberating oscillations of energy loops generated through the strings’ oscillatory motion cause potentiation of weak synchronizations of change. The oscillatory motion generated simultaneously through the inner and outer layer of strings transmits to mind normal matter. Thus information transformed by mind a FPDM acts as input and executive signals, distributed through the EMR. Mind normal matter takes action based on the degree of an oscillating difference its circuitry receives. The information then received by mind normal matter through EMR gets transformed through circuitry. These events represent preconscious activity that precedes cognitive activity through ‘mind’ observing ego.

IV ‘Mind’ observing ego

*Embodied states of mind represent autopoiesis: a system capable of reproducing and maintaining biological information*.

‘Mind’ observing ego in this theory is synonymous with the more familiar term individual. It is a viewpoint of consciousness that defines conscious events but is not consciousness in and of itself [2]. ‘Mind’ observing ego is an emergent property of consciousness that occurs through the unconscious activity of mind a FPDM of a particular embodied state. This relationship represents a ‘strange loop’ and bidirectional system: it functions in two directions through a closely maintained connection existing. As previously discussed, this connection exists fundamentally through events in the macrocosm that include the oscillatory motion of some closed strings on the inner layer of compacted multi-dimensional space. A pure mental state is distinctly expressed in embodied states because mind FPDMs is recognizably different from something else: mind dark energy of a similar type. A similar type here refers to a type of change that occurred through the mind’s infinite potential. When manifested, infinite potential caused mind dark energy in the macrocosm to emerge and included the potential to transform the state of pure awareness. Potential differences then occurred when mind dark energy morphed through dynamic duality as the open and closed dark energy strings oscillated in the ground state. Thus, emerged mind FPDMs that (through the oscillatory motion of some closed strings on the inner layer of compacted multi-dimensional space) generated momentum. Events follow that occur in the microcosm through embodied states of mind. Duality in the microcosm manifests in embodied states through open and closed oscillating strings attached to a dark energy circle that creates an inner and outer layer of mind a FPDM.

Biological information is a transformative process that occurs through the active circuitry signals of mind normal matter and dark energy strings’ oscillatory motion of mind a FPDM. This interdependent relationship through which biological information occurs manifests dynamically as ‘difference’ but with ‘sameness.’ The ‘sameness’ fundamentally through just one reference point: signals through which physiological systems regulate and where no physiological bond exists between mind a FPDM of different embodied state. ‘Mind’ observing ego of each embodied state perceives dynamic differences with a sense of being an individual because of ‘sameness’ fundamentally through a foundation of interoceptive patterns (i.e., patterns of bodily sensations) ‘from within.’ This process occurs through the oscillatory motion of strings of mind a FPDM acting as signals that trigger a wave of activation and ‘sameness’ as Feelings of Knowing (FOKs) interoceptive patterns perceived by ‘mind’ observing ego. Because of this bidirectional relationship, it is difficult to prove the complete absence of consciousness. Not all unconscious events of mind a FPDM are alike, and features of sensory processes such as highly practiced automatisms through 'mind' observing ego do not apply to unconscious events. Through FOKs interoceptive patterns that do not deviate much from ‘day-to-day,’ ‘mind’ observing ego of a particular embodied state bridges the gap between the mental and physical domains by defining conscious events but does so with ‘sameness.’

*The actions and reactions of mind a FPDM ‘bend back on’ and affect an overall self-reflective cognitive process of ‘mind’ observing ego.*

Included in this process is the perception of excitatory and inhibitory responses. Cognitive actions are promoted or prevented through reactions [24]. Cognition emerges through the responses of ‘mind’ observing ego as reactions capable of producing effects of which there are three types of reactions: reflexive, habitual, and volitional:

A. Reflexive reactions: An unconditioned response with an assumed degree of automaticity and a high degree of reflexivity. Reflexive reactions create an intuitive sense of position and motion state in physical space via the embodied internal state and proprioceptive senses.

B. Habitual reactions: A combined response as a reflexive reaction followed by an impulsive reaction (a sudden reaction without planning and impulse action). Habitual reactions represent an over-practiced response experienced with a high degree of having little mental choice due to reflexivity.

C. Volitional reactions: A conditioned response with a degree of an agency; thus, things can be caused to happen. There are two volitional reactions: An impulsive reaction and a reaction with forethought.

*Cognition is a process that begins with instructions received through ‘mind’ observing ego as bottom-up encoded signals.*

As previously discussed, when patterns of dynamic difference are detected through conical EMR, this causes mind a FPDM to capture small amounts of ‘data’ to create ‘atoms.’ These ‘atoms’ once generated , are converted when stimuli get mapped to create ‘bottom-up’ encoded input signals and where unconscious events encode conscious expectations about sensory input causes [22]. ‘Mind’ observing ego perceives bottom-up encoded signals initially as primordial feelings. These feelings represent cognitive ‘atoms’ and ‘building blocks’ for conceptual and nonconceptual mental content. Accordingly, through an unconditioned response, differences are perceived coherently as primordial feelings that act as stimuli that cause ‘mind’ observing ego to interpret and process change. ‘Mind’ observing ego discerns automatically primordial feelings as pleasant, unpleasant, or neutral. It remembers the difference as Feelings of Knowing (FOKs). Through FOKs, a certainty is created but with little descriptive detail as feelings of familiarity, feelings of rightness and wrongness, or feelings of beauty and goodness [2].

Accordingly, through the perceptions of ‘mind’ observing ego, FOKs are cognized as vague judgments, intuitions, abstract concepts, and vivid expectations [2]. These FOKs do not fade even with ‘disuse,’ nor do they fade over time; access to them does fade somewhat [25]. FOKs act as memory network activation. The change that makes a difference as primordial feelings and FOKs compete for the attention of ‘mind’ observing ego that learns experientially through three types of reactions (e.g., reflexive, habitual, and volitional). As part of the initial unconditioned responses of ‘mind’ observing ego to bottom-up encoded signaling is the spontaneous creation of the ‘primordial sense of self’ that occurs through FOKs interoceptive patterns.

Interoceptive inference regulates bodily states [22] and occurs through the attention and reflexive reactions of ‘mind’ observing ego. This interoceptive inference becomes the foundation for embodied ‘selfhood’ experienced by ‘mind’ observing ego that is a robust set of concepts critical for the sense of embodiment, motivation, and wellbeing [26]. The foundation for ‘real-world cognition’ of ‘mind’ observing ego that includes experience and observation occurs through interoception (i.e., bodily sensations). With a foundation of interoception, ‘mind’ observing ego creates an internal map as the ‘sense from within’ that acts as a goal directing stimuli with two immediate cognitive tasks [23]:

A. Determine whether the received bottom-up encoded input signals match the expectation of what that information should be.

B. Determine if the stimulus signals a state of affairs that is potentially threatening or rewarding.

Through the attention and reactions of ‘mind’ observing ego, interoceptive predictions are made across a perceptual processing hierarchy. Interoceptive predictions occur by way of two fundamental mechanisms [22]:

A. The bottom-up mechanism through attention and reflexive reactions occurs once encoded signals are received and create input expectations as sensory input experience.

B. The top-down mechanism through attention and volitional reactions; impulsive reaction alone or impulsive reaction followed by reaction with forethought and create interoceptive predictions. These predictions then compete with bottom-up encoded input expectations.

Every top-down prediction is reciprocated with a bottom-up prediction error, ensuring that interoceptive predictions act as constraints through sensory information [22]. Prediction error [22] is the difference between:

A. Encoded bottom-up signals received and cognitively processed through ‘mind’ observing ego as sensory information input expectations.

B. Comparison of that input through ‘mind’ observing ego with top-down predictions fundamentally as FOKs interoceptive patterns. This mechanism ensures that the incoming encoded bottom-up signals received as sensory input continuously interact with higher-order cognitive representations.

Because every top-down prediction is reciprocated with a bottom-up prediction error, the viewpoint of ‘mind’ observing ego includes a sense of self with motivational context as goals, history, and environment [27]. ‘Mind’ observing ego through active inference, thus attention and volitional reactions, resolves sensory prediction errors about the state of the body in two key ways [22]:

A. Updates top-down predictions to make them more like the bottom-up input expectations. Thus, sensory input is experienced as bottom-up encoded.

B. Resolves prediction errors, and bottom-up input expectations become more like top-down predictions. Thus, sensory input is experienced in an alternative way than was bottom-up encoded.

In general, the reactions of ‘mind’ observing ego through primordial feelings and FOKs interoceptive patterns cues a response as a ‘searchlight’ of attention [28]. This reaction/attention sequence acts as an action that plays a prominent role in binding [29] change that makes a difference. Through chunking, organization, and learned associations [2], ‘mind’ observing ego discerns the content of conical EMR cognitively. The reactions of ‘mind’ observing ego suppress proprioceptive prediction error by changing proprioceptive sensations [22]. This suppression rests on proprioceptive predictions, fulfilled by attention and response as reflexive reactions. The volitional reactions and perception of ‘mind’ observing ego through cognitive appraisals of interoceptive patterns related to embodied states inform as response selection [26]. Thus, minimized is the same prediction error [30]. Moreover, the current concerns of ‘mind’ observing ego are mostly unconsciously driven by mind a FPDM [2].

*The mental processes of ‘mind’ observing ego can be envisioned through numbered connected components with interoception as a substrate of mental content.*

**Figure 12.**
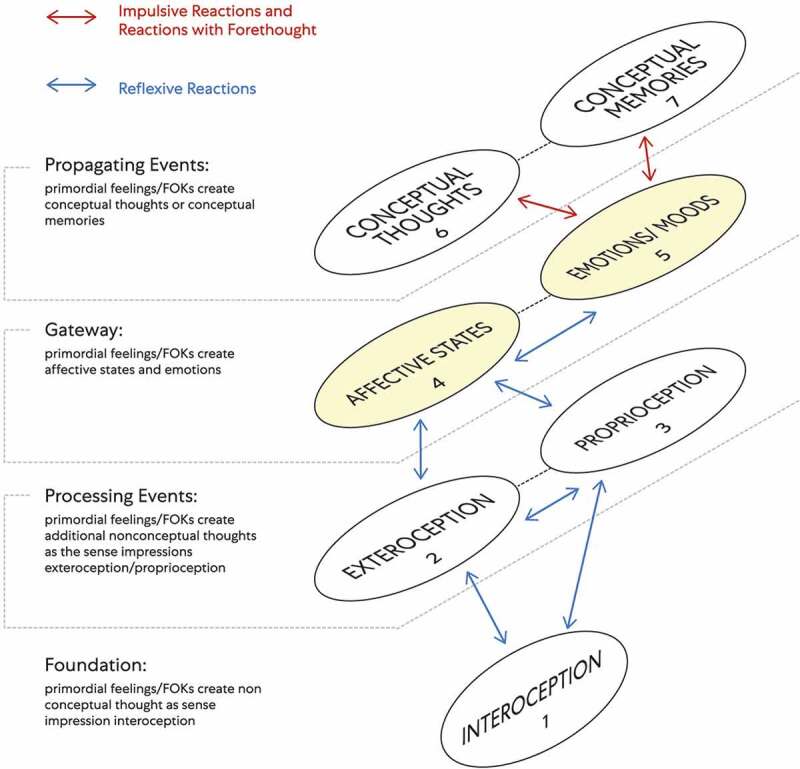
‘Mind’ observing ego/Avatar living being with a brain

The components map model presented is a way to envision the mental processes of ‘mind’ observing ego (see Figure 12). Mental processes consist of nonconceptual mental content and conceptual mental content.

1. Nonconceptual mental content is based on the theory that some mental states can represent the world of appearances even though the individual possessing those states’ does not have the concepts required to specify their content [31].
2. Conceptual mental content is defined here as thinking based on abstract cognitive processes: dealing with ideas rather than events that include fundamental units of thought (e.g., true or false, believed, desired, hoped for) from an individual’s perspective [31]. Included is conceptualization, or the process of forming a concept of something that includes analytical or problem-solving ideas and symbols (a mark, word, or sign that through mental process links an idea, ‘object,’ or relationship).

The different ‘layers’ of biological information related to cognition are depicted with interoception as a substrate of mental content. When the Avatar living being has a brain (e.g. invertebrates, mammals, birds, amphibians, reptiles, and fishes), ‘mind’ (observing ego) experiences emotions and makes interoceptive predictions across a hierarchy of perceptual processing through attention and reactions. Blue arrows depict reflexive reactions: A unconditioned response with an assumed degree of automaticity and a high degree of reflexivity. Red arrows depict impulsive reactions: a sudden reaction without planning and impulse action and reactions with forethought: those reactions that are more considered and entail forethought.

This model sets out the different ‘layers’ of cognitive processes through numbered connected components with interoception as a substrate of mental content. Each numbered component represents a subsystem (i.e., a self-contained system within a more extensive system) with causal relationships. One subsystem can affect other subsystems, and a ‘ripple effect’ can spread through the whole system.

Definition of Terms (that correlate with the model’s numbered components)

Nonconceptual Components of the Components Map Model

1. Interoception: relates to stimuli produced within an embodied state perceived as bodily sensations ‘from within.’ These sensations through conscious experience act as the foundation for embodied ‘selfhood’ critical for the sense of embodiment, motivation, and wellbeing [26].

2. Exteroception: the faculty of sensation and sensitivity to stimuli originating ‘outside’ an embodied state. This sensation faculty includes an ability to see, hear, touch, smell, taste, and sense pain [2].

3. Proprioception: the awareness of position and movement through an embodied state.

4. Affective States: states that refer to psycho-physiological constructs. These states include nonconceptual concepts (e.g., exteroception and proprioception) through a foundation of (e.g., pleasant, unpleasant, or neutral) FOKs interoceptive patterns. Through affective states, mental and physical processes vary along these principal dimensions: valence (i.e., pleasant or unpleasant, positive or negative), arousal, and motivation (i.e., through the desire to procure or avoid bodily sensations). Accordingly, through awareness of FOKs interoceptive patterns, affective states vary in duration, intensity, specificity, and arousal level [32]. Neutral affective states may go unnoticed, or ‘mind’ observing ego may recognize them due to pleasant/positive or unpleasant/negative valence associated with interoceptive patterns.

An affective state in this model acts as an antecedent to both emotion and mood. Faster onset and shorter duration of affective states are associated with emotion, and a slower-onset, longer duration of affective states are associated with mood [33]. The difference between emotion and mood fundamentally: an emotion is associated with a more intense interoceptive pattern. Emotion is thus more likely to be provoked by an affective state through intense bodily sensations. A mood is associated with a less intense interoceptive pattern. Mood is thus less likely to be provoked by an affective state through intense bodily sensations. Instead, a mood is likely to be provoked by an affective state through a less intense bodily sensation. Patterns of bodily sensations create the feeling tone [34] of an affective state. An effective state’s feeling tone is the immediate and spontaneous affective experience of interoceptive awareness.

Conceptual Components of the Components Map Model

5. Emotions or emotion–like states: in their broadest sense are states characterized by loosely coordinated changes as related to these following aspects [33]:

A. Feeling– changes in subjective experience through primordial feelings and FOKs. Interoceptive patterns encode into affective feelings [35]. Examples; FOKs interoceptive patterns as nociception (continuous bodily sensation patterns experienced with a decreased tolerance to pain), antinociception (continuous bodily sensation patterns experienced with increased tolerance to pain), disgust (a rush of unpleasant bodily sensation patterns that quickly subside), orgasm (a rush of pleasant bodily sensation patterns that quickly subside).

B. Cognition: changes in attentional, perceptual, and inferential processes (appraisals).

C. Action: changes in the predisposition for or execution of specific responses through the three reactions (i.e., reflexive, habitual, and volitional). Reactions in this model are the action (active) components of cognition.

D. Expression: changes in the facial, vocal, and postural appearance of a closely related Avatar living being.

E. Physiology: changes in physiological activity that may include neural activity. Every emotion is associated with a physiological experience. For example, the emotion of fear through interoceptive patterns is often associated with a faster heart rate and muscle tensing, and possible shortness of breath [368].

Here are some examples of emotions; happiness, sadness, fear, anger, surprise, embarrassment, jealousy, guilt, and pride [37].

6. Conceptual Thoughts [2]:

A. perceptual stimuli (e.g., inner speech, dreams, visual imagery)

B. fleeting present

C. autobiographical episodes (experienced)

D. expectations and effortful voluntary control

E. explicit beliefs (about ‘self’; about the world)

F. novel skills, abstract concepts

7. Conceptual Memories [2]:

A. fading traces of present in immediate memory

B. autobiographical episodes (recalled)

Components 1–5 represent cognition that exists through an unconditioned response as processing events. Therefore, cognition is created with an assumed degree of automaticity, a high degree of reflexivity, where 1-4 is nonconceptual mental content. An affective state acts as an antecedent that cues an emotion or mood, and because of this, the same nonconceptual content can be conceptual. An affective state can be nonconceptual as a perceptual state or conceptual as part of an emotional state’s contents [38]. Affective states and emotions demarcate a layer that acts as a gateway to conceptual mental content: conceptual thoughts and conceptual memories as propagating events.

The sequence of affective states followed by emotions represents unconditioned responses that collectively act as ‘conditioned stimuli.’ ‘Mind’ observing ego experiences emotion with an immediate and spontaneous transformation of a reflexive reaction to an impulsive reaction through a habitual reaction. An impulsive reaction is the first volitional response after experiencing emotion; before this, the reactions of ‘mind’ observing ego are reflexive. Through a combined response as a habitual reaction, ‘mind’ observing ego after an emotion experiences a high degree of reflexivity. The habitual reaction is an over-practiced response. An impulse (i.e., urge) accompanies the emotion experienced [36]. This urge acts as a motivation to procure or avoid bodily sensations. If not overridden by a volitional reaction with forethought, ‘mind’ observing ego ‘self actualizes’ interoceptive motivation through conceptual mental content. The more sensitive ‘mind’ observing ego is to bodily sensations, the more difficult it is to initiate a reaction with forethought after experiencing the associated emotion. In this way, ‘mind’ observing ego often responds to intense interoceptive patterns associated with emotion with certain kinds of ‘over thinking’ behavior. In this model, ‘free will’ implies volitional reactions of ‘mind’ observing ego constrained through varying interoceptive sensitivity degrees.

‘Mind’ observing ego decides on and commits to a particular course of action through volitional reactions. In this model, volitional reactions that follow habitual reactions cause ‘objects’ of conceptual mental content to serially link, thus create the perception of motion: forward motion through impulsive reactions or reversed motion through reactions with forethought. Higher-level cognition can affect reactivity on a semi-permanent basis to interoceptive cues ultimately. Through a habitual reaction sequence, ‘mind’ observing ego ‘thinks reality’ either as component 6 (conceptual thoughts) or component 7 (conceptual memories). This scenario represents conditioned responses and where the conceptual thought or conceptual memory acts as ‘unconditioned stimuli.’ Accordingly, affective states followed by emotions as ‘conditioned stimuli’ connect in various ways to conceptual mental content as ‘unconditioned stimuli. Interoceptive patterns associated with emotion represent unconditioned responses that are acquired over time and create ‘sameness.’ This ‘sameness’ generalizes to multiple contexts as conceptual thoughts or memories that become progressively more accessible through conditioned responses. Through ‘bottom-up’ expectations that compete with ‘top-down’ predictions, ‘mind’ observing ego will either accept or reject encoded signaling. Reality as higher-level cognition occurs through two fundamental processing sequences:

1. Affective states through interoceptive patterns cue emotion with a degree of automaticity, a high degree of reflexivity, and emotions are correlated with ‘objects’ of conceptual mental content.
2. Affective states through interoceptive patterns cue emotion with a degree of automaticity, a high degree of reflexivity, and interoceptive patterns are correlated with ‘objects’ of conceptual mental content.

According to this theory, ‘mind’ observing ego while perceiving with a human viewpoint connects the mental and physical domains by defining conscious events and potentially can create eight different types of competencies through cognition [to varying degrees, 39]:

1. Logical/Mathematical

2. Linguistic

3. Musical

4. Spatial

5. Bodily-Kinesthetic

6. Naturalist

7. Interpersonal

8. Intrapersonal

V Avatar living being

*An Avatar living being is a ‘visual’ indicator of emergent intelligence processes of biological information.*

When ‘mind’ observing ego experiences conceptual mental content (e.g., conceptual thoughts or memory) later in cognitive construction, it often includes a body: the physical structure concept of a living being that may include bones, flesh, and organs. An Avatar living being is a ‘visual’ indicator of emergent intelligence processes of biological information of a particular embodied state. However, it does not contain ‘mind’ observing ego, and nothing in that of ‘mind’ observing ego contains an Avatar living being. Nevertheless, experience shows that ‘mind’ observing ego and a particular Avatar living being are closely related. Through the FOKs interoceptive patterns,’ it is ‘mind’ observing ego rather than the Avatar living being that derives a ‘sense of self.’ When conceptual mental content gets linked through the reactions of ‘mind’ observing ego, the closely related Avatar living being moves in varied directions. Avatar living beings have distinctive characteristics that include a brain (e.g., invertebrates, mammals, birds, amphibians, reptiles, and fishes) and those without a brain (e.g., slime mold and plants).

The relative dimensions of reality as thought always connect to the deeper dimensions of the Universe/conscious concept and apply here to the relative question: Do nonhuman species as Avatar living beings experience emotion and mood? According to this model, the answer is no; they do not, however, nor do humans as Avatar living beings experience emotion. Instead, it is ‘mind’ observing ego and not the Avatar living being that experiences anything, including emotion. When the closely related Avatar living being has a brain, ‘mind’ observing ego experiences emotion. Thus, it can ‘suffer’ through interoceptive patterns associated with emotion ultimately. In this theory, suffering includes degrees of interoceptive sensitivity associated with emotion and mood experienced through ‘mind’ observing ego, and where the closely related Avatar living being has a brain. Through interoceptive patterns associated with a wide range of emotions that include dysregulation and interoceptive abnormalities, ‘mind’ observing ego suffers mentally and physically. Cognition research related to animals fundamentally focuses on questions about the mechanisms involved in specific capacities: learning, memory, perception, or decision-making, including an investigation into animal concepts, beliefs, and thoughts [40]. According to this theory, the Avatar living being displays emergent intelligence processes as behavior and ‘mind’ observing ego is inferred through comparative cognition. The suffering of ‘mind’ observing ego is correlated in innumerable ways through the ‘visual’ indicator Avatar living beings with a brain. Suffering in its broadest sense is characterized by loosely coordinated changes as related to these following core interoceptive dimensions:

1. Valence: pleasant/positive or unpleasant/negative.

2. Arousal: to stimulate action through reactions.

3. Motivation: the desire to procure or avoid bodily sensations.

**Figure 13.**
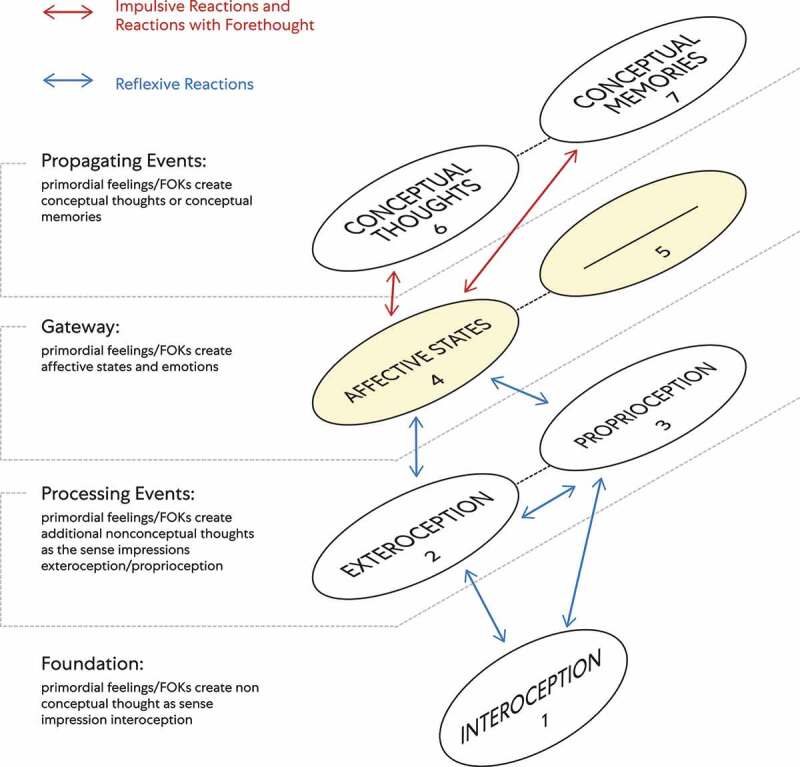
‘Mind’ observing ego/Avatar living being without a brain

When the closely related Avatar living being is without a brain (e.g., slime mold and plants), ‘mind’ observing ego does not experience emotion but can initiate volition through reactions. Accordingly, the closely related Avatar living being does not display behavior indicative of ‘mind’ observing ego experiencing suffering (see Figure 13).

The different ‘layers’ of biological information related to cognition are depicted with interoception as a substrate of mental content. When the Avatar living being is without a brain (e.g. slime mold and plants), ‘mind’ observing ego does not experience emotions but can make interoceptive predictions across a hierarchy of perceptual processing through attention and reactions. Blue arrows depict reflexive reactions: A unconditioned response with an assumed degree of automaticity and a high degree of reflexivity. Red arrows depict impulsive reactions: a sudden reaction without planning and impulse action and reactions with forethought: those reactions that are more considered and entail forethought.

*The Universe and consciousness are about change, and change is a story about reactions that can be understood through the fundamental elements’ relationship.*

Either narrowly or broadly defined, dynamic differences have beginnings and ends through interdependent relationships that condition each other. The mind, a fundamental entity without beginning or end, does not change ultimately; however, with infinite potential, the change that made a difference occurred. Potential energies that converted or transformed caused dynamic differences. Through interdependent relationships, dynamic differences have occurred and are occurring in innumerable ways. Each embodied state of mind represents a cyclic although not repetitive journey thus can be construed as a “Hero’s Journey” [41]: a story of change that includes struggle, inner and outer obstacles, and meaningful transformation. The mind is not in an embodied state per se; instead, it perceives the consciousness produced through a viewpoint as ‘mind’ observing ego. Arguably, ‘mind’ observing ego rather than the Avatar living being is closest to representing the image of the mind without beginning or end, albeit with a cognitive viewpoint. Thus, the term “I,” as related to this theory, stands for ‘mind’ observing ego rather than a closely related Avatar living being. Implied is that the inconceivable aspects of the mind that correlate with the Universe/consciousness concept, when conceptualized, equates to ‘unable to be seen’ and thus ‘I’ is a term synonymous with an ‘invisible’ viewpoint as ‘mind’ observing ego.

Through interplaying fundamental elements, consciousness dynamically evolves as distinguishable differences. Part of the dynamics that dictate how consciousness evolves includes ‘mind’ observing ego beginning its life of cyclic existence with a total loss of memory, thus amnesic-like to its connection to the mind. However, the loss is temporary, but the time it takes for ’mind’ observing ego, i.e. ‘hero,’ to regain memory through volitional reactions is variable. In this theory, the Universe and consciousness through an interplay between fundamental elements give rise to the phenomena “Spooky action at a distance,” self-governing through strings conversion and cyclic existence where innumerable consciousnesses as dynamic differences that come to an end inevitably lead to a universal ‘Big Crunch.’ The discussion section of this article concludes with these phenomena.

“*Spooky action at a distance” represents a process through a substrate and interdependent relationships.*

With interoception acting as a substrate of mental content and through a close relationship with an Avatar living being ‘mind’ observing ego experiences disease and death and can infer ‘others’ presence. Through FOKs interoceptive patterns that do not deviate much from ‘day-to-day,’ ‘mind’ observing ego senses itself as an individual, and thus different from ‘other’ mind observing egos of different embodied states. Because mind dark energy with the state of pure awareness acts as the substrate for embodied states, an interoceptive network exists. Through the interoceptive network, a phenomenon occurs termed “Spooky action at a distance”: a concept that an ‘object’ can move, change or is affected without being physically touched by another ‘object.’ According to this theory, the ‘objects’ are conceptual mental content perceived by ‘mind’ observing ego, with a cognitive viewpoint, that infers through thought acts of observation, and measurement.

‘Mind’ observing ego, with a cognitive viewpoint, makes a logical judgment about ‘objects’ based on circumstantial evidence and prior conclusions rather than direct observation. Action may be deemed ‘spooky’ because pure awareness, the state of mysterious mind dark energy, cannot be explained as a qualitative difference in Western thought. Through the interoceptive network, however, different consciousnesses are cognitively connected and where learning takes place experientially. When access to consciousness produced by a particular embodied state is authorized, ‘mind’ observing ego engages in ‘hacking,’ and relationships unfold straightforwardly or undoubtedly. When access is unauthorized, mind observing ego is a ‘hacker’ and unfolds relationships that are entangled: complicated or questionable. Nevertheless, through this mental activity, the mind through ‘mind’ observing ego participates interactively in numerous consciousnesses simultaneously. According to this model, “Spooky action at a distance” represents a process explainable through a substrate and interdependent relationships:

1. Interdependent relationships occurring through the substrate, pure awareness as homogeneous intelligent information of dark energy where signals get exchanged instantaneously. The information then acts as instantaneous input signals received through mind FPDMs with a purely mental state.

2. Interdependent embodied relationships occurring through the substrate primordial/proto-consciousness/unconscious intelligence as patterned intelligent information, where information is received as instantaneous input signals. These signals are processed through mind a FPDM in relationship with mind normal matter. Thus, mind FPDMs receive input signals from mind dark energy but with a dynamic process that differs from receiving and exchanging information with mind normal matter.

3. Interdependent embodied relationships occurring through the substrate, conscious events’ intelligence as intelligent information of patterned conscious events, where information gets exchanged between mind normal matter and mind FPDMs through EMR. Thus physiological systems are regulated as signals, including developmental processes through a developmental program encoded by mind a FPDM with degrees of evolutionary conservation. However, a delay occurs through EMR between the moment information gets sent and received.

4. Interdependent embodied relationships occurring through the substrate cognitive intelligence and interoception as a substrate of mental content. The information encoded by mind a FPDM is defined through 'mind' observing ego as conscious events with delay as inference: a cognitive process and a conclusion reached by ‘mind’ observing ego based on evidence and reasoning. When biological functions with degrees of evolutionary conservation are defined by 'mind' observing ego, through cognition, this is depicted as the behavior and appearance of the closely related Avatar living being and other Avatar living beings.

‘Mind’ observing ego through inference and the concept of “Spooky action at a distance” mentally bridges the ‘gap’ between different information exchange networks. Through ‘sameness’ existing fundamentally through a foundation of FOKs interoceptive patterns ‘from within,’ reality’s dynamic differences are perceived with a sense of being an individual. Because of no physiological connection existing, ‘mind’ observing ego perceives differences as ‘others’ ultimately. When the closely related Avatar living being has a brain, ‘mind’ observing ego as a ‘hacker’ or when ‘hacking’ experiences a post-interoceptive phase (i.e., empathetic or apathetic). Through feelings of affective states that cue associated emotion or mood, this phase occurs, and after ‘mind’ observing ego experiences FOKs interoceptive patterns continuously or as a rush that quickly subsides where habitual reactions follow. As previously discussed, through affective feelings as nociception, antinociception, disgust, or orgasm with an associated positive or negative emotion or mood, ‘mind’ observing ego connects impulsively nonconceptual mental content to conceptual mental content. In this way, conceptual thought or conceptual memory includes the reality of ‘others’ that occurs with a post interoceptive phase of either:

A. Post-interoceptive phase empathic: the ability to understand and share the feelings of another.

B. Post-interoceptive phase apathetic: a lack of interest, enthusiasm, or concern that includes another’s feelings.

S*elf-governing relates fundamentally to the process where the viewpoint of* ‘*mind*’ *observing ego evolves from purely an egocentric mental state to purely an altruistic mental state; both bottom-up encoded by mind FPDMs.*

Through situations termed the macrocosm and the microcosm, interdependent events and ‘strange loops’ occur. Regarding the consciousness of embodied states, the role of unconscious processing events through mind normal matter and mind FPDMs is to generate information, including the regulation of physiological systems through signals and developmental processes with degrees of evolutionary conservation. Through ‘mind’ observing ego, the role of conscious events is to either accept or reject the information generated through volitional reactions that causes feedback to occur. Thus influences the processing of mind a FPDM and equates to ‘self- governance’ as part of the autopoiesis of embodied states [42]. According to this theory, the biologically relevant concept of information includes 'mind' observing ego that through defining conscious events gives 'meaning' to biological functions encoded by mind a FPDM.

Embodied states define the capacities of ‘mind’ observing ego that through volitional reactions learn experientially and corresponds with the appearance of the closely related Avatar living being. When ‘mind’ observing ego through volitional reactions begins to reject primitively egocentric bottom-up processing, more evolved altruistic top-down processing occurs. Interoceptive patterns are integral to higher-order cognition through an essential role in regulating ‘mind’ observing ego, which affects the mind a FPDM through string conversion. Accordingly, through interoceptive patterns acting as cues, flexibility in this relationship is lower or higher [43] expressed as a greater or lesser capacity to carry out tasks. In turn, strings converted have consequences related to cyclic existence. A lack of flexibility in the relationship correlates with less potential for cognition: knowledge and understanding through thought, experiences, and the senses as an attribute of consciousness. Alternatively, flexibility in relationship correlates with more potential for cognition to emerge as an attribute of consciousness.

The more sensitive ‘mind’ observing ego is to bodily sensations, the more difficult it to initiate a reaction with forethought after experiencing an associated emotion. ‘Mind’ observing ego often becomes ‘inflamed’ through attachment or aversion to interoceptive patterns that cue emotion integral to the imagery process. This idea may be related to connectionist theories where learning is achieved when an individual can form associations between a particular stimulus and response. ‘Free will’ implies volitional reactions constrained through varying degrees of interoceptive sensitivity. Through volitional reaction, ‘mind’ observing ego is free to choose action and react impulsively or override it through a reaction with forethought. However, the capacity to do this fundamentally depends on the sensitivity of ‘mind’ observing ego to FOKs interoceptive patterns. In this way, ‘mind’ observing ego often responds to intense interoceptive patterns associated with emotion through habitual reactions where higher-level cognition is fundamentally experienced as bottom-up encoded. Thus, the viewpoint of ‘mind’ observing ego includes varying degrees of knowledge and understanding and where current concerns can be summarized here as: ‘me,’ ‘myself,’ and ‘I.’ However, when a volitional impulsive reaction is willingly transformed into a reaction with forethought as a consequence, practice ensues. Thus, ‘mind’ observing ego experientially begins the process of learning how to anchor its reactivity [25]. This process comes from both rejection and acceptance of bottom-up encoded processing that may be experienced as prolonged conceptual thought combined with concentration where ‘mind’ observing ego masters the ability to stay focused nonconceptually on a single subject without getting distracted. In this way, ‘mind’ observing ego learns to ‘tame’ mind a FPDM and where bottom-up encoded signaling no longer surprises or injures because through a reaction with forethought, for example, FOKs unpleasant interoceptive patterns associated with emotion (e.g., fear) is willingly experienced. Thus, the viewpoint of ‘mind’ observing ego changes over time through varying degrees of knowledge and understanding that can be summarized here as ‘I’ stands for ‘you.’

Although numerous, only through one fundamental entity as the mind without beginning or end does ‘mind’ observing ego exist ultimately. Thus, all viewpoints and the closely related Avatar living beings represent fundamentally ‘the same kind of different.’ However, between relationships that condition each other occurring, Time and ‘others’ stands in the duo [44] as a stubbornly persistent illusion in the viewpoints of many mind observing egos of embodied states.

*When signals of volitional reactions are received and ‘recorded’ by mind a FPDM as strings’ conversion, this acts as ‘cyclic controls’ through embodied states of mind.*

In this theory, innumerable consciousnesses begin in the same way, i.e., a particular mind a FPDM that through the ‘strange loop’ and bidirectional relationship causes ‘mind’ observing ego to emerge as the viewpoint of consciousness of an embodied state. As previously mentioned, ‘mind’ observing ego begins its life of cyclic existence with a total loss of memory of its connection to the mind. Moreover, the closely related Avatar living being is without a brain. From this starting point, consciousness evolves, and the proposed system of autopoiesis includes enabled strings’ conversion. Although the exact number of strings attached to a circle of compacted multi-dimensional space is unknown, mind FPDMs interacting with mind normal matter for the first time in the microcosm will have an equal number of closed and open strings. Strings are not destroyed, and the number of strings neither increases nor decreases. Instead, the enabled open and closed strings of mind FPDMs convert from open to closed or closed to open. As previously discussed, the consciousness produced in embodied states is depicted as conical EMR related to electromagnetism. Volitional reactions represent the action component of cognition with an agency’s degree, thus the cognitive control of ‘mind’ observing ego. Through volitional reactions of ‘mind’ observing ego that causes feedback to mind a FPDM, complex warping in the spheroidal region (tiny black hole) occurs.

The volitional reactions of ‘mind’ observing ego cause the conical EMR to get pulled into a tiny black hole. The EMR then distorts and representing ‘a transferred property’ as fixed energy it propagates. Thus, in the circle of compacted multi-dimensional space, consciousness moves within an electromagnetic field before transferring to attached open and closed strings. Not destroyed, the enabled strings of mind a FPDM instead convert from open to closed or closed to open. A momentary transformation in an embodied relationship through the production of the fixed energy of EMR gets expressed as photons: the electromagnetic field’s quantum, including electromagnetic radiation. This description redefines the term blackbody radiation that traditionally refers to the spectrum of light emitted by any heated object. In this theory, blackbody radiation occurs through ‘a focal point’ as a circle of dark energy, thus ‘body’ of mind FPDMs.

Conical EMR that gets pulled into a tiny black hole in this model implies a momentary end of a cognitive cycle. Thus, when conical EMR gets pulled into a tiny black hole, a particular end occurs, and through this relationship, impermanence is implied. Each mind a FPDM with strings that convert acts as the ‘record keeper’ [30] and data storage process that mirrors changes in each embodiment. The conversion of enabled strings acts as the record of oscillatory motion left by the current volitional reactions of ‘mind’ observing ego and what they have been. Thus the evolutionary role through string conversion can be understood as a sort of 'informational domain' that exists alongside the domain of matter and energy.

In this way, oscillatory motion represents ‘oscillating cosmic dust’: a small movement distributed through interdependent relationships with cyclic consequences. ‘Mind’ observing ego while hacking’ or acting as a ‘hacker’ and where the presence of ‘others’ is inferred through the interoceptive network often incurs ‘debt.’ The volitional reactions of ‘mind’ observing ego cause suffering of ‘others’ fundamentally through the interoceptive network and acts of inference. Thus, the ‘debt’ incurred is through volitional reactions of ‘mind’ observing ego of a particular embodied state that are often correlated with acts of the closely related Avatar living being. This causes the conversion of enabled strings of mind a FPDM that acts as the ‘cosmic ledger’ of the debt incurred. The debt recorded as enabled string conversion will be ‘paid-up’ experientially in a current embodied state or a future embodied state due to cyclic existence. The theory’s proposed autopoiesis and ‘self’ governance through string conversion is represented. However, string conversion is not a sadistic form of ‘self’ governance where ‘mind’ observing ego of a particular embodied state that suffered ‘wins’ when pain, suffering, or humiliation is inflicted on a particular ‘mind’ observing ego that caused the suffering. Instead through enabled string conversions, a mechanism necessary for change gets ‘set in motion.’ To ‘walk a mile in someone else’s shoes’ means that before ‘an individual is judged, the judge must understand others’ experiences, challenges, and thoughts.’ This admonition applies to the mind that through the experiences of ‘mind’ observing ego of particular embodied states, ‘enlightened’ consciousness can occur ultimately. Accordingly, the mind will only be motivated to make changes once it knows the suffering experientially through the viewpoint ‘mind’ observing ego. Implying ignorance is rectified experientially through cyclic existence and part of autopoiesis and ‘self’ governance where the suffering experienced as the ‘victim’ motivates the change. The number of strings that convert is based on the motivation of ‘mind’ observing ego received through feedback signals to mind a FPDM.

A. The lack of willingness to override an impulsive reaction or reaction with forethought for the sake of ‘self’ causes closed strings to convert to open strings.

B. The willingness to override an impulsive reaction or reaction with forethought for the sake of ‘others’ causes open strings to convert to closed strings.

C. When the reaction is reflexive, thus an unconditioned response with an assumed degree of automaticity and a high degree of reflexivity, no strings convert.

*The Universe and consciousness have always coexisted; thus, laws can be infinite in Time and complexity.*

The Universe and consciousness have always coexisted, governed by interdependent relationships expressed through the laws of cause and effect [30]. These laws emerging in conceptual thought are associated with the timing of the emergence of ‘mind’ observing ego through innumerable embodied states amid a chain of events in constant transformation. Thus, laws can be infinite in Time and complexity. Through the very tight coupling existing as feedback between mind a FPDM and reactions of ‘mind’ observing ego the concept of Time is produced as a conceptual mental construct in many different ways. Time represents a dynamical system that is dependent on a function that describes an event through a point in a geometrical space:

1. The function: volitional reactions of ‘mind’ observing ego act as a function.

2. The event: the motion of enabled open and closed strings acts as an oscillatory event.

3. The point: a dynamic change as a point in oscillatory motion that occurs through dark energy strings’ conversion.

4. Geometrical space: a circle of compacted multi-dimensional space of mind FPDMs.

*When the consciousnesses produced through a particular embodied state as a dynamic difference comes to an end, cyclic existence occurs.*

Each embodied state of mind has a unique timeline, and because of this, the number of embodied relationships in the microcosm increases or decreases accordingly. Death is an event that marks the end of a particular consciousness, and birth is an event that represents a beginning. Both are often traumatic events. According to this theory, ‘mind’ observing ego that has not prepared for the event of death often does not experience this event ‘peacefully.’ Instead, death is often experienced with fear due to a relationship connection continuing while others are ending. Some connections are fundamental to cyclic existence that represents a system of ‘cyclic autopoiesis’:

1. The connection between the mind as a fundamental entity that is not entirely conceivable without beginning or end and mind dark energy with the copresent state of pure awareness.

2. The connection between mind dark energy and mind FPDMs with the copresent state pure mental.

3. The connection between mind FPDMs and mind normal matter with copresent state mental images.

4. The connection between mind FPDMs and ‘mind’ observing ego with a cognitive mental state.

5. The connection between ‘mind’ observing ego and an Avatar living being.

At death, the connection between mind a FPDM and ‘mind’ observing ego of a particular embodied state exists. However, the connection between mind a FPDM and mind normal matter with copresent state mental images is severed. Moreover, the connection between ‘mind’ observing ego and the closely related Avatar living being is severed. Death of individual consciousness is a scenario with cyclic implications, and what comes into play is the result of ‘debt’ incurred. Thus includes instances while ‘mind’ observing ego was hacking or acted as a ‘hacker’ through the interoceptive network. The consequences of the actions of ‘mind’ observing ego will have to be dealt with eventually is the rule of the Universe/consciousness concept according to this theory that occurs through interdependent ‘connected’ relationships ultimately. Thus, implied through a proverb: “What goes around comes around.”

When a particular embodied relationship ends, mind normal matter transforms into its previous form mind normal energy, present but in an indistinct state of mind rather than expressed mental images. Due to the abundance of embodied relationships in the microcosm, non-synchronized interactions begin and end where mind normal energy is in high demand. When mind normal energy is unavailable, there will be mind FPDMs in an intermediate state. Perhaps best understood through the terms microcosm and macrocosm, this state is a situation where ‘mind’ FPDMs is demarcated from mind dark energy through the oscillatory motion of dark energy strings in the ground state but without a framework of mind normal matter. As previously described, when mind normal energy is available, mind as FPDMs without a framework with a position on the Universe’s dark energy substrate generates momentum with the excited state of some closed strings. Cyclic embodied relationships occur through gravity as an invisible consequence that pulls mind normal energy toward mind FPDMs. Cyclic changes manifest through the consciousness of an embodied state that includes mind a FPDM with a purely mental state modified through oscillatory motion and conversion of two string types.’ The inner and outer layers of strings are not destroyed. Instead, they are converted from open to closed or closed to open, and are carried by mind a FPDM through a circle of compacted multi-dimensional space. Thus mind a FPDM represents the bearer of consequences that include how mind normal matter gets configured. As previously described, through the oscillatory motion of strings as contact forces, a transformative mechanism occurs. In the microcosm, additional transformative mechanisms occur in part through mental images as the state and fundamental difference between mind normal energy and mind normal matter. In turn, because of closely maintained connections existing, produced is the emergent viewpoint ‘mind’ observing ego with the perception of being an individual. This viewpoint is linked to a closely related Avatar living being that will be unique and differs from previous embodiments.

Consciousness evolves with differences that include appearance and character through a framework mind normal matter, a cognitive viewpoint ‘mind’ observing ego, and a closely related Avatar living being. Flexibility through enabled strings, consciousness, and cognition represents the potential for ‘mind’ observing ego of a particular embodiment to emerge with a viewpoint that is both just and fit to judge having acquired due to cyclic existence varying degrees of knowledge and understanding. Thus, a process where a certain continuity is maintained through the relationship and ‘strange loop’ between mind a FPDM and ‘mind’ observing ego. Through varying degrees of volitional reaction (i.e., more reaction-more strings convert, less reaction-fewer strings convert) because of attachment or aversion fundamentally to procure or avoid bodily sensations 'mind' observing ego caused string conversion. Through embodied states and cyclic occurrence of both rejection and acceptance of bottom-up encoded processing, the viewpoint of ‘mind’ observing ego reveals more or less cognitive flexibility: the ability to adapt behaviors in response to changes in an embodied environment.

*Because consciousness and the Universe are bonded by relationship, “Every beginning has an end” is a statement that applies both to individual consciousnesses and the collapse of the Universe in the ‘Big Crunch’ event.*

The Big Bang(s) may represent the start of this cycle, which is one of an infinite number of cycles of interactions between mind FPDMs and mind normal matter. The cycles may stop, however, as a consequence of gravity. Instead of universal expansion, there may be a Big Crunch in which the macrocosm collapses into itself. As previously described, in the macrocosm, gravity is a consequence instead of a force that brings together interdependent relationships of a dynamic change that makes a difference. However, in the microcosm through, EM waves of a particular embodied state get pushed toward the circle of compacted multi-dimensional space of mind a FPDM. Implied in this theory is that through gravity, a dynamic difference as the Universe/consciousness concept reaches a particular end. “Every beginning has an end” is a statement that applies to individual consciousnesses created by a particular embodied state. The ending of particular embodied states overtime collectively causes the collapse of the Universe and the ‘Big Crunch’ event ultimately. Thus, consciousness and the Universe are bonded by relationship.

The relative dimensions of reality always connect to deeper dimensions and apply to the Universe/consciousness concept’s unsettling aspects. Part of the dynamics that dictate how consciousness evolves includes ‘mind’ observing ego through volitional reactions choosing to cause harm by acts of inference. ‘Mind’ observing ego through the interoceptive network can encroach, infiltrate, slaughter, rape, and dominate [45]. Why? Because fundamentally ‘mind’ observing ego is sensitive to varying degrees of interoceptive patterns. Until ‘mind’ observing ego learns how to anchor its reactivity to interoceptive patterns associated with emotions and moods, the mind, through innumerable viewpoints, will suffer ultimately. Thus, as a melding of consciousness of embodied states, reality will continue to ‘play out’ with acts of atrocities displayed as avatars living beings’ behavior that emerge in the Universe/consciousness by way of conceptual thoughts or memories.

Although beyond the scope of this article, strings that demarcate mind a FPDM from mind dark energy can potentially dissolve into a circle of compacted multi-dimensional space through a particular population of strings (e.g. one open and the rest closed). Accordingly, ‘Endings’ are derived through a process from a particular type of oscillatory pattern of all closed strings but one open string on circles of compacted multi-dimensional space. When such events occur through innumerable circles of compacted multi-dimensional space, this dynamically adjusts the macrocosm conditions. ‘Mind’ observing ego as part of this process ultimately knows it exists experientially through transformation into one entity, mind dark energy. Thus, ‘mind’ observing ego ends its life of cyclic existence with a total gain of memory. Eventually, as the process of transformation reverses, the macrocosm that includes mind dark energy and mind normal matter/normal energy absorbs into isotopic space. The mind, a fundamental entity without beginning or end, does not change ultimately; however, dynamic change may occur with infinite potential existing. Thus, a new cycle of energies that make dynamic differences through interdependent relationships can potentially manifest.

## Conclusion

‘Relative truth’ as defined by this theory is the usual way ‘mind’ observing ego, with a human cognitive viewpoint, perceives the world of appearances. Arguably discrepancies must exist while ‘mind’ observing ego tries to express its intuitions as best it can about the reality it perceives. In general, empirical evidence occurs when ‘mind’ observing ego of many different embodied states agree about observations. This process explains how what was once ‘scientific fact’ is now ‘science fiction,’ and arguably not because of more advanced technologies. Instead, ‘mind’ observing ego with a closely related Avatar that is a human being has advanced its ability to define conscious events more complexly. In the end, the innumerable different viewpoints arguably are facets of the same reality. The main emphasis of the article is to provide intriguing explanations. With that intent, there is the potential to ‘rethink’ relationships that set up pleasant/positive, unpleasant/negative relationship valence:’ predator-prey,’ or ‘friend-enemy’ or ‘family-outsiders.’ As part of a model presented in this article, suffering includes degrees of interoceptive sensitivity associated with emotion and mood that occurs through ‘mind’ observing ego, and where the closely related Avatar living being has a brain. Although beyond this article’s scope, the author argues that a radical shift in ‘thinking’ that includes nonhuman species’ ethics and the moral consideration of animals and how nonhuman animals ought to be treated is in order. For scientific discovery to move forward, ‘mind’ observing egos as thinking individuals must transcend the ordinary and thus understand many different things, not just one. According to this theory, one of the greatest errors of ‘mind’ observing ego is its lack of understanding that it is a viewpoint of consciousness rather than the closely related Avatar living being. This fundamental lack of understanding leads to confusion, such as belief in a solid reality to what it perceives and that the world of appearances has strict rules of cause and effect. Part of what needs to be added into the equation that allows ‘mind’ observing ego to tackle the complex problems that are the reality these days and where the closely related Avatar living being is a researcher, is understanding the distinction between ‘self’ and ‘others’ is an illusion of conceptual mental content.

The ‘correctness’ of a theory arguably is merely a measurement of how well it correlates with other ‘accepted’ theories and its capacity to explain areas of knowledge. This article represents a transdisciplinary unifying theory with a framework created by the fundamental elements that can be summarized by combining the italicized statements found within the article’s discussion section. The Origin of the Universe is a science story about what happens when the mind’s infinite potential manifests change. The mind without a beginning or end with infinite potential is not entirely conceivable. Because the mind exists without beginning or end, unobservable reality existed before the ‘Big Bang.’ The microcosm represents a set of circumstances when mind FPDMs becomes embodied by mind normal matter, and when the conceptual designation related to the story’s location changes, a symbolic ‘Big Bang’ occurs. The circumstances occurring in the microcosm can be envisioned through the concepts of a ‘body,’ ‘cosmic brain,’ and framework. Consciousness occurs in the microcosm and is due to a cooperative condition. For a cooperative condition to occur, the strings of mind FPDMs need to change from the ground state to the excited state. Information received from mind normal matter gets transformed similarly through the oscillatory motion of enlisted open and closed strings that form an inner layer. Enlisted open and closed strings that form an outer layer reactively change oscillatory motion from the ground state to the excited state and thus transform information received and when they are ‘triggered.’ Through the inner oscillatory string layer, physiological systems regulate, and thus ‘sameness’ is created as a reference point. Gravity in the microcosm is a ‘push force,’ and EM waves pushed toward the center of a dark energy circle create a pattern envisioned as a centralized tiny black hole. When pushed toward the tiny black hole, EM waves bend and configure into conical EMR that in this model represents the form of consciousness produced through embodied states.

An underlying intelligence associated with the pure mental state of mind a FPDM transmits from the envisioned tiny black hole and in a way that ‘illuminates’ conical EMR. Mental images represent underlying conscious events’ intelligence of mind normal matter that has differences through circuitry’s oscillatory motion. Embodied states of mind represent autopoiesis: a system capable of reproducing and maintaining biological information. The actions and reactions of mind a FPDM ‘bend back on’ and affect an overall self-reflective cognitive process of ‘mind’ observing ego. Cognition is a process that begins with instructions received through ‘mind’ observing ego as bottom-up encoded signals. The mental processes of ‘mind’ observing ego can be envisioned through numbered connected components with interoception as a substrate of mental content. An Avatar living being is a ‘visual’ indicator of emergent intelligence processes of biological information. The Universe and consciousness are about change, and change is a story about reactions that can be understood through the fundamental elements’ relationship. “Spooky action at a distance” represents a process through a substrate and interdependent relationships. Self-governing relates fundamentally to the process where the viewpoint of ‘mind’ observing ego evolves from purely an egocentric mental state to purely an altruistic mental state; both bottom-up encoded by mind a FPDM. When signals of volitional reactions are received and ‘recorded’ by mind a FPDM as strings’ conversion, this acts as ‘cyclic controls’ through embodied states of mind. The Universe and consciousness have always coexisted; thus, laws can be infinite in Time and complexity. When the consciousnesses produced through a particular embodied state as a dynamic difference comes to an end, cyclic existence occurs. Because consciousness and the Universe are bonded by relationship, “Every beginning has an end” is a statement that applies both to individual consciousnesses and the collapse of the Universe in the ‘Big Crunch’ event.

## References

[1] The Feynman Lectures on Physics-Caltech. The Feynman Lectures on Physics, Volume 1 Chapter 4. Conservation of Energy [Internet]. Pasadena, CA: California Institute of Technology. 1970. Available from: https://www.feymannlectures.caltech.edu/I_toc.html

[2] Ricard M, Thuan Trinh X. International society for science and reli-gion. In: The quantum and the lotus : a journey to the frontiers where science and bud-dhism meet. Cambridge: International Society For Science And Religion. New York, NY: Three Rivers Press; 2007.

[3] Giammarchi, Marco G. Review of ELEMENTARY PHILOSOPHICAL AND THEOLOGICAL CONSEQUENCES OF QUANTUM MECHANICS. Eur J Sci Theol. 2015;11(3):47–59.

[4] Wilczek F. A beautiful question : finding nature’s deep design. New York: Penguin Books; 2016.

[5] MIT School of Engineering Can brain waves interfere with radio waves? (2011). Mit Engineering. https://engineering.mit.edu/engage/ask-an-engineer/can-brain-waves-interfere-with-radio-waves/

[6] Bohm D. A new theory of the relationship of mind and matter. Philos Psy-chol. 1990;3(2–3):271–286.

[7] Katrin. B, Schwarz, John H, Becker. M. String theory and m-theory : a modern introduction. Cambridge: Cambridge University Press; 2007.

[8] NASA. Dark Energy, Dark Matter/Science Mission Directorate. Nasa.gov, 2012, science.nasa.gov/astrophysics/focus-areas/what-is-dark-energy

[9] McMullin E. The Origins of the Field Concept in Physics. Phys Perspect. 2002;4(1):13–39.

[10] Kibble T. Englert-Brout-Higgs-Guralnik-Hagen-Kibble mechanism (history). Scholarpedia. 2009;4:1.

[11] Klebanov IR, Mal-dacena JM. Solving quantum field theories via curved spacetimes. Phys Today. 2009;62(1):28–33.

[12] Silberstein M, Stuckey WM, Mcdevitt T. Beyond the dynamical universe : unifying block universe physics and time as experienced.England: Oxford University Press; 2018.

[13] Zieffler A, Garfield J, Delmas R, et al. (2016). (2008) A framework to support research on informal inferential reasoning 5. Available from: http://www.stat.auckland.ac.nz/~iase/serj/SERJ7 (2)_Zieffler.pdf

[14] Budday S, Raybaud C, Kuhl E. A mechanical model predicts morphological abnormali-ties in the developing human brain. Sci Rep. 2015;4(1):5644. 10.1038/srep05644 PMC4090617 25008163

[15] Godfrey-Smith P, Kim S.Biological information.The stanford ency-clopedia of philosophy (Summer 2016 Edition).Edward N, Zalta.editors.https://plato.stanford.edu/archives/sum2016/entries/information-biological/.

[16] Sloman A 2018 “HOME PAGE: AARON SLOMAN.” Www.Cs.Bham.Ac.Uk.. http://www.cs.bham.ac.uk/~axs.

[17] Rancourt L, Tattersall PJ. Further experiments demonstrating the effect of light on gravitation. Appl Phys Res. 2015;7(4):4.

[18] Liboff AR. Magnetic correlates in electromagnetic consciousness. Electro-magnetic Biol Med. 2016;35(3):228–236. 10.3109/15368378.2015.1057641 27049696

[19] Vlach HA, Sandhofer CM. Fast mapping across time: memory processes support children’s retention of learned words. Front Psychol. 2012;3. DOI:10.3389/fpsyg.2012.00046(19) 22375132 PMC3286766

[20] Seth AK, Friston KJ. Active interoceptive inference and the emotional brain. Philos Trans R Soc B. 2016;371(1708):20160007. 10.1098/rstb.2016.0007 PMC5062097 28080966

[21] Smythies J, Edelstein L, Ramachandran V. Hypotheses relating to the function of the claustrum. Front Integr Neurosci online. 2012;6:53. Available at: . 22876222 10.3389/fnint.2012.00053 PMC3410410

[22] Ziemann U, Hallett M, Cohen LG. Mechanisms of deafferentation-induced plasticity in human motor cortex. J Neurosci. 1998;18(17):7000–7007. 9712668 10.1523/JNEUROSCI.18-17-07000.1998 PMC6792971

[23] Pollard-Wright H. Electrochemical energy, primordial feelings and feelings of knowing (FOK): mindfulness-based intervention for interoceptive experience related to pho-bic and anxiety disorders. Med Hypotheses. 2020;144:109909. 32505847 10.1016/j.mehy.2020.109909

[24] Farb N, Daubenmier J, Price CJ, et al. Interoception, contemplative practice, and health. Front Psychol online. 2015;6:6. Available at:: . 26106345 10.3389/fpsyg.2015.00763 PMC4460802

[25] Damasio A. Mental self: the person within. Nature. 2003;423(6937):227. 12748620 10.1038/423227a

[26] Crick FC, Koch C. A framework for consciousness. Nat Neurosci. 2003;6(2):119–126. 12555104 10.1038/nn0203-119

[27] Vohn R, Fimm B, Weber J, et al. Management of attentional resources in within-modal and cross-modal di-vided attention tasks: an fMRI study. Hum Brain Mapp. 2007;28(12):1267–1275. 17315224 10.1002/hbm.20350 PMC6871278

[28] Rab-Gsal-Zla-Ba, Dil-Mgo Mkhyen-Brtse, Matthieu R, et al. The collected works of Dilgo Khyentse. Boston: Shambhala; 2010. p. 409.

[29] Bermúdez J, Arnon C.Nonconceptual mental content.The stanford encyclo-pedia of Philosophy (Summer 2020 Edition).Edward N, Zalta.editors.https://plato.stanford.edu/archives/sum2020/entries/content-nonconceptual/.

[30] Frijda NH. Emotion Experience and its Varieties. Emotion Rev. 2009;1(3):264–271. Available at:. online.

[31] Paul ES, Sher S, Tamietto M, et al. Towards a comparative science of emotion: affect and consciousness in humans and animals. Neurosci-ence Biobehav Rev. 2020;108:749–770. 10.1016/j.neubiorev.2019.11.014 PMC6966324 31778680

[32] Grabovac, Andrea D, Lau MA, Willett BR. Mechanisms of mindfulness: a buddhist psychological model. Mindfulness. 2011;2(3):154–166.

[33] Pace-Schott EF, Amole MC, Aue T, et al. Physiological feelings. Neurosci Biobehav Rev. 2019;103:267–304. 31125635 10.1016/j.neubiorev.2019.05.002

[34] Barlow, David H. Unified protocol for transdiagnostic treatment of emotional disor-ders : workbook. New York, NY: Oxford University Press; 2018.

[35] Damasio A. 2000 10. Feeling of what happens: body and emotion in the making of consciousness, Mariner Books, pp. 1.

[36] Jr. H, Richard G. Nonconceptual content and the ‘space of reasons.’. Philos Rev. 2000;109(4):483.

[37] Gardner H. Frames of Mind : the Theory of Multiple Intelligences. New York: Basic Books; 1985.

[38] Andrews K. 2008. “Animal Cognition.” Plato.Stanford.Edu, 1. https://plato.stanford.edu/archives/sum2016/entries/cognition-animal/

[39] Campbell J. The hero with a thousand faces. Novato, Calif: New World Li-brary; 2008. p. 17.

[40] Eelen P, Hermans D, Baeyens F. Learning perspectives on anxiety disorders. In: E. J. L, C. D, J., editors. Anxiety disorders: an introduction to clinical management and research. New York: Wiley; 2001. p. 249–264.

[41] Rateliff N (2020). Nathaniel rateliff - time stands (official audio) [YouTube Video]. In YouTube. https://www.youtube.com/watch?v=1rZGCDJRAJU

[42] Burgin M, Cárdenas-García JF. A dialogue concerning the essence and role of information in the world system. Information. 2020;11(9):406

